# Ca^2+^ administration prevents α-synuclein proteotoxicity by stimulating calcineurin-dependent lysosomal proteolysis

**DOI:** 10.1371/journal.pgen.1009911

**Published:** 2021-11-15

**Authors:** Lukas Habernig, Filomena Broeskamp, Andreas Aufschnaiter, Jutta Diessl, Carlotta Peselj, Elisabeth Urbauer, Tobias Eisenberg, Ana de Ory, Sabrina Büttner

**Affiliations:** 1 Department of Molecular Biosciences, The Wenner-Gren Institute, Stockholm University, Stockholm, Sweden; 2 Department of Biochemistry and Biophysics, Stockholm University, Stockholm, Sweden; 3 Institute of Molecular Biosciences, University of Graz, Graz, Austria; 4 BioTechMed Graz, Graz, Austria; 5 Field of Excellence BioHealth–University of Graz, Graz, Austria; National Centre for Biological Sciences, TIFR, INDIA

## Abstract

The capacity of a cell to maintain proteostasis progressively declines during aging. Virtually all age-associated neurodegenerative disorders associated with aggregation of neurotoxic proteins are linked to defects in the cellular proteostasis network, including insufficient lysosomal hydrolysis. Here, we report that proteotoxicity in yeast and Drosophila models for Parkinson’s disease can be prevented by increasing the bioavailability of Ca^2+^, which adjusts intracellular Ca^2+^ handling and boosts lysosomal proteolysis. Heterologous expression of human α-synuclein (αSyn), a protein critically linked to Parkinson’s disease, selectively increases total cellular Ca^2+^ content, while the levels of manganese and iron remain unchanged. Disrupted Ca^2+^ homeostasis results in inhibition of the lysosomal protease cathepsin D and triggers premature cellular and organismal death. External administration of Ca^2+^ reduces αSyn oligomerization, stimulates cathepsin D activity and in consequence restores survival, which critically depends on the Ca^2+^/calmodulin-dependent phosphatase calcineurin. In flies, increasing the availability of Ca^2+^ discloses a neuroprotective role of αSyn upon manganese overload. In sum, we establish a molecular interplay between cathepsin D and calcineurin that can be activated by Ca^2+^ administration to counteract αSyn proteotoxicity.

## Author summary

The accumulation and aggregation of neurotoxic proteins represents a hallmark of age-associated neurodegenerative disorders. Mostly, this is accompanied by a reduction of the cell’s capacity to proteolytically remove these aggregation-prone proteins. Thus, stimulation of degradative pathways to clear neurotoxic proteins represents an emerging theme to counteract neurodegeneration. The pathology of Parkinson’s disease (PD) is intimately connected to α-synuclein aggregation, and α-synuclein mutations or increased α-synuclein protein levels upon gene duplication cause hereditary PD. Using simple model systems to study α-synuclein toxicity, we establish a novel regime that re-activates cellular degradative capacity and prevents α-synuclein-induced cellular decline. Specifically, we show that increasing the bioavailability of Ca^2+^ stimulates protein degradation within the lysosome, the cell’s waste bin and recycling facility. Whereas α-synuclein compromised cellular Ca^2+^ homeostasis and reduced the activity of the lysosomal protease cathepsin D, simple administration of extra Ca^2+^ corrected these defects. We provide insights into the molecular pathways underlying cytoprotection achieved by Ca^2+^ supplementation and identify a causal role for central calcium signaling pathways in Ca^2+^-mediated stimulation of cathepsin D activity. In sum, our results establish a regime to improve the cellular capacity to cope with proteotoxic stress that functions across species barriers and might be transferable to other neurotoxic proteins.

## Introduction

Parkinson’s disease (PD) is a progressive neurodegenerative disorder strongly associated with age and characterized by the selective degeneration and loss of dopaminergic neurons in the *substantia nigra pars compacta* [1,2]. Neuronal dysfunction during PD is coupled to the formation of intracellular protein inclusions termed Lewy bodies, mainly composed of α-synuclein (αSyn) [3]. The etiology of PD is assumed to be multifactorial, involving genetic susceptibility, aging and environmental risk factors such as heavy metals and pesticides as drivers of the disease [2]. The association between neurological damage and disrupted metal ion homeostasis has been established decades ago, and numerous epidemiological studies indicate that exposure to distinct metals, in particular iron, manganese, copper, and zinc, represents a clear risk factor for PD [4–7]. As transition metals serve as essential cofactors for a plethora of metalloproteins and thus impact biological processes at all levels, any perturbation of metal ion homeostasis will compromise cellular functionality. This is particularly evident in the brain, an organ that accumulates metal ions. Here, a disequilibrium of metal ions has been suggested to progressively disrupt Ca^2+^ homeostasis and in consequence essential neuronal functions that depend on tightly regulated cytosolic Ca^2+^ levels [8–10]. In line, the aggregation of αSyn as a main factor of both sporadic and familial PD [11–15], is intimately linked to metal ion homeostasis in general and to imbalances in Ca^2+^ homeostasis in particular: several metal ions, including Ca^2+^, have been shown to directly induce conformational changes of this intrinsically disordered protein and can accelerate aggregation and fibrillation of αSyn as well as cell-to-cell transmission [16–19]. *Vice versa*, αSyn impacts on metal ion homeostasis, in particular cellular calcium (Ca^2+^) handling and sequestration [20]. The interrelation between Ca^2+^ homeostasis and neuronal demise associated with αSyn remains enigmatic and seems highly dependent on the cellular context. Binding of Ca^2+^ to αSyn can induce the formation of αSyn oligomers both *in vivo* and *in vitro* [21–24], and αSyn has been shown to increase cellular influx and cytosolic levels of Ca^2+^, which in turn might amplify its oligomerization [25–29]. Furthermore, cleavage of αSyn by the Ca^2+^-activated protease calpain modulates its cytotoxicity [30,31]. Interestingly, binding of Ca^2+^ to αSyn has been proposed to regulate the physiological function of this protein chameleon that can adopt various structural conformations: although αSyn itself has only a moderate affinity for Ca^2+^, this seems physiologically relevant in cellular environments characterized by high Ca^2+^, such as pre-synaptic terminals. Here, binding of Ca^2+^ to the negatively charged C-terminus of αSyn has been shown to increase its lipid-binding capacity, enabling αSyn-mediated vesicle clustering and tethering of vesicles to the plasma membrane [21]. How αSyn causes a pathological increase of cytosolic Ca^2+^ is still under debate, but might involve increased membrane permeability, allowing influx of extracellular Ca^2+^ or altering intracellular storage capacity, or a direct interaction with organellar Ca^2+^ transporters, for instance the sarcoendoplasmic reticulum Ca^2+^ ATPase (SERCA) [32–35]. Similarly, it remains unclear how disrupted Ca^2+^ homeostasis connects to other pathological changes caused by excess or mutated αSyn, including for instance impaired proteostasis. Sub-optimal activity of different proteostatic subsystems, including autophagy and general lysosomal function, has been repeatedly linked to neurodegeneration associated with αSyn [36–39], and lysosomal Ca^2+^ has been suggested to impact the clearance of proteotoxic aggregates [40].

To further elucidate the connection between αSyn proteotoxicity and metal homeostasis in general as well as disrupted Ca^2+^ homeostasis in particular, we employed yeast and Drosophila expressing human αSyn, two genetically amenable model systems successfully used to unravel basic mechanisms of PD-associated cellular dysfunction [41–45]. We demonstrate that αSyn selectively increases total cellular Ca^2+^ content, while levels of other metals remain unchanged. Surprisingly, external administration of Ca^2+^ prevented αSyn proteotoxicity in yeast and Drosophila models. Mechanistically, external Ca^2+^ administration provided cytoprotection via stimulation of the lysosomal protease Cathepsin D and required functional calcineurin signaling. In sum, our data suggest that dietary Ca^2+^ supplementation impacts distinct aspects of the cellular proteostasis system, which concertedly counteract the proteotoxic consequences of αSyn.

## Results

### Administration of external Ca^2+^ protects against αSyn proteotoxicity

**Fig 1:**
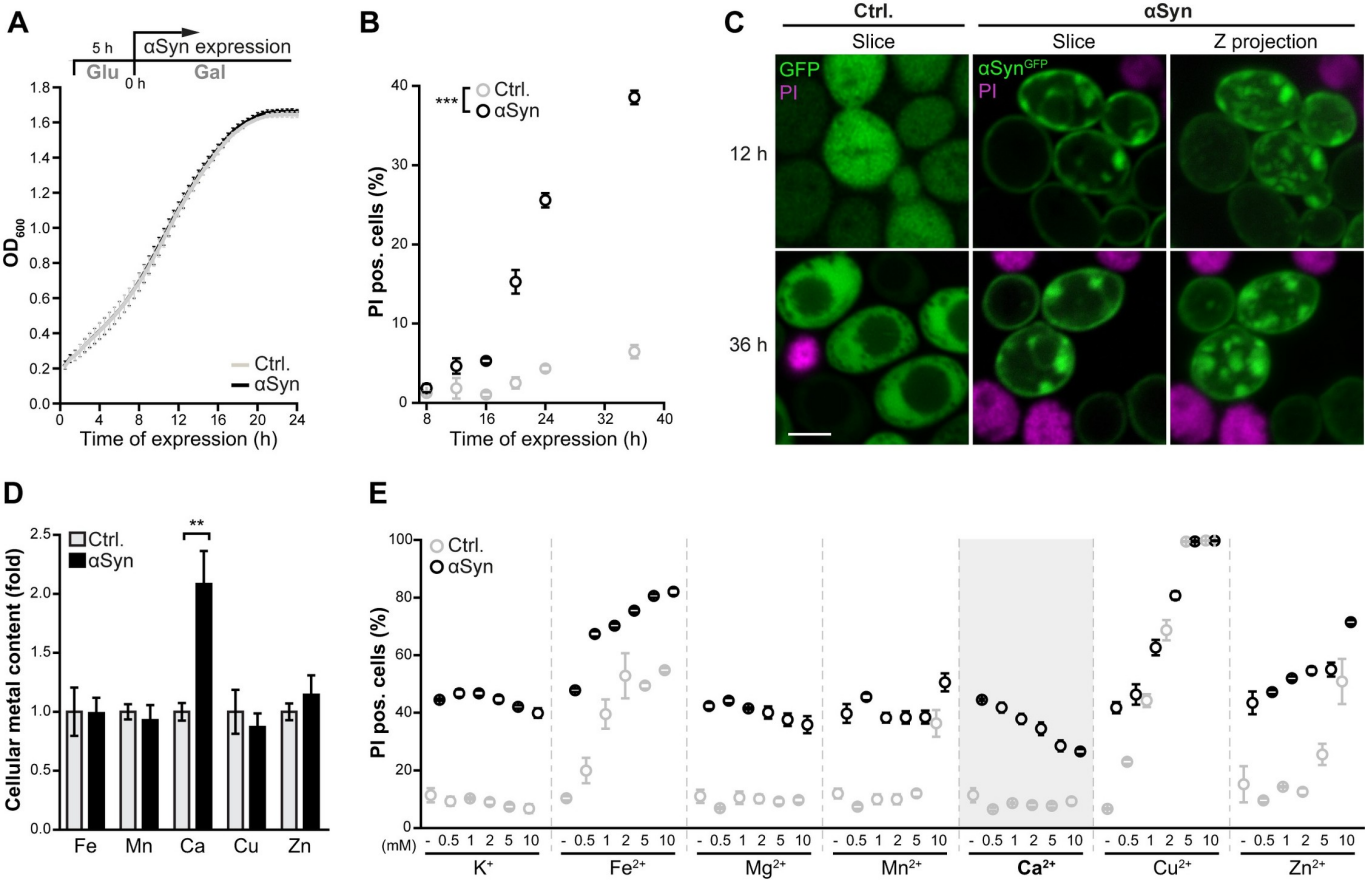
Administration of external Ca^2+^ protects against αSyn proteotoxicity. (**A**) Growth kinetics of wild type cells expressing human α-synuclein (αSyn) or harboring the corresponding vector control (Ctrl.) upon shift to galactose for promoter induction. Optical density (OD_600nm_) was determined every 30 min. Means ± s.e.m; n = 4. (**B**) Flow cytometric quantification of loss of membrane integrity via propidium iodide (PI) staining in cells expressing αSyn or harboring the vector control at indicated time points. Means ± s.e.m; n = 5. (**C**) Confocal micrographs of cells expressing GFP or αSyn^GFP^ for 12 h and 36 h. Cells were counterstained with PI to visualize dead cells. Z-projections of three-dimensional stacks as well as a representative section are shown. Scale bar represents 2 μm. (**D**) Total cellular metal content (Fe, Mn, Ca, Cu, Zn) of cells expressing αSyn for 24 h quantified via total reflection X-ray fluorescence (TXRF). Values were normalized to vector control cells per metal. Means ± s.e.m; n = 4. (**E**) Flow cytometric quantification of cell death via propidium iodide (PI) staining of cells expressing αSyn for 36 h or harboring the vector control. Growth medium was supplemented with indicated concentrations of K^+^, Fe^2+^, Mg^2+^, Mn^2+^, Ca^2+^, Cu^2+^ or Zn^2+^. Means ± s.e.m; n = 4. **p<0.01 and ***p<0.001.

To analyze the link between disrupted metal homeostasis and αSyn proteotoxicity, we first assessed how αSyn would impact cellular metal content and, *vice versa*, how metal overload would affect αSyn toxicity employing a humanized budding yeast model. Most neurons are in a non-dividing, post-mitotic state. Thus, we used an experimental setup in which the xenotopic expression of human αSyn driven by a galactose promoter did not impact proliferation (Fig 1A) but instead triggered cellular dysfunction upon prolonged incubation in a post-mitotic state. Entry into the stationary phase (starting from ~20–24 h of culture time) coincided with a prominent increase of αSyn-induced cell death, as demonstrated by flow cytometric quantification of propidium iodide (PI) staining, indicative of lethal membrane integrity loss (Fig 1B). High levels of αSyn killed about 40% of the cell population within 36 h of incubation, while control cells displayed less than 10% dead cells (Fig 1B). Confocal microscopic analysis of the subcellular distribution and aggregation behavior of an αSyn-GFP chimera revealed no obvious differences between proliferating and post-mitotic cells (12 h *versus* 36 h). As reported previously [45], αSyn decorated the plasma membrane and appeared in small, membrane-attached as well as larger, cytoplasmic inclusions (Fig 1C). We used total reflection X-ray fluorescence (TXRF) spectrometry to quantitatively map the impact of αSyn on total cellular metal content after 24 h of cultivation. Interestingly, αSyn selectively increased total Ca^2+^ levels, while all other metals were unaffected (Fig 1D). Like mammalian cells, yeast cells ensure low resting cytosolic Ca^2+^ levels in the range of 50–200 nM through the action of an array of different Ca^2+^ channels and transporters facilitating organellar sequestration [46–48]. The prominent increase of total cellular Ca^2+^ levels in the presence of αSyn suggests enforced assimilation and subsequent sequestration into organellar Ca^2+^ stores, as efficient decoding of temporal Ca^2+^ signals necessitates a low resting cytosolic Ca^2+^ level [46].

As the overexposure to different metals is linked to αSyn-induced cellular dysfunction, we next assessed whether increased environmental levels of metal ions would impact on αSyn proteotoxicity. The concentrations of different metal ions used to study their effect on αSyn oligomerization, aggregation and cytotoxicity, both *in vitro* and *in vivo*, cover a rather broad range (from μM to mM) [19,49–52]. Aiming for intracellular overload of metal ions, we challenged cells with 500 μM—10 mM of several divalent cations and the monovalent cation K^+^ and quantified cell death after 36 h of αSyn expression and metal ion exposure. Though excess Fe^2+^, Zn^2+^ and in particular Cu^2+^ killed cells in a concentration-dependent manner, this was not specific for αSyn expression but reflected general metal ion poisoning *per se* (Fig 1E). Rather surprisingly, administration of Ca^2+^ prevented αSyn-induced cell death. This cytoprotection was dosage-dependent and Ca^2+^-specific, as Mg^2+^, another alkali earth metal with quite similar chemical and physical properties, had no effect (Fig 1E). Thus, though αSyn prominently disrupted Ca^2+^ homeostasis, causing cells to assimilate and sequester Ca^2+^ way beyond the physiological level, a further increase of Ca^2+^ availability in the surrounding still decreased αSyn toxicity. In model systems ranging from yeast, nematodes and flies to human cell culture and mice, αSyn toxicity has been linked to increased cytosolic Ca^2+^ levels [25,26,28,29], though molecular details remain unclear. Hence, any cytoprotective effect of Ca^2+^ administration seems rather counterintuitive.

### An external Ca^2+^ pulse enforces organellar ion storage and mitigates the αSyn-driven increase of cytosolic Ca^2+^

**Fig 2:**
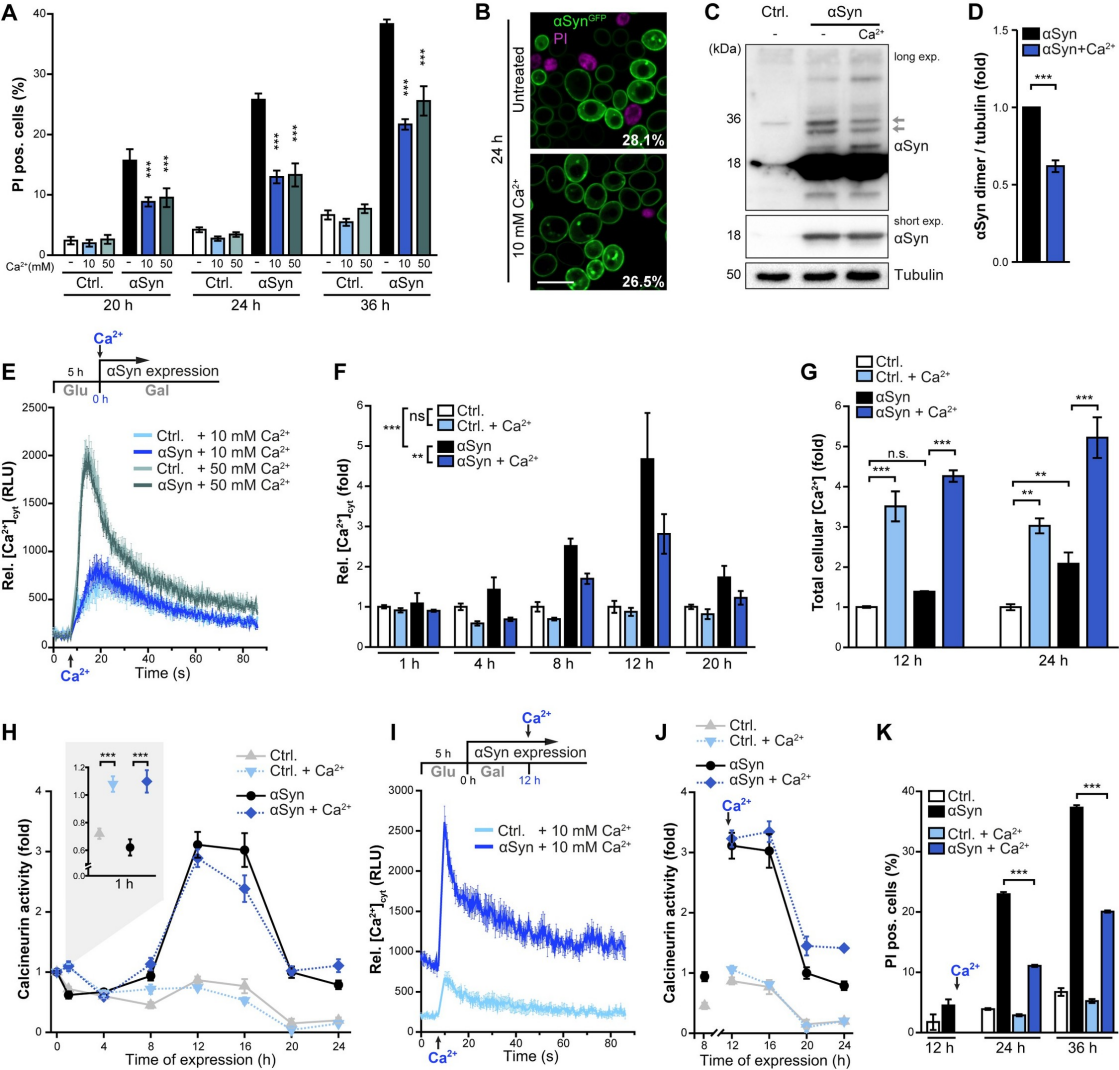
An external Ca^2+^ pulse enforces organellar ion storage and mitigates the αSyn-driven rise in cytosolic Ca^2+^. (**A**) Flow cytometric quantification of cell death via propidium iodide (PI) staining of cells expressing αSyn for indicated time points or harboring the vector control (Ctrl.). Cells were supplemented with additional 10 mM and 50 mM CaCl_2_ at the time point of shift to galactose or left untreated. Means ± s.e.m; n = 8. (**B**) Confocal micrographs of cells expressing αSyn^GFP^ for 24 h counterstained with PI to visualize dead cells. Cells were supplemented or not with 10 mM Ca^2+^ at the time point of shift to galactose media. Scale bar represents 5 μm. Values indicate mean percentages of cells with αSyn^GFP^ aggregates, with the following s.e.m.: *Untreated*: 28.1% ± 1.5% (in total 395 cells were evaluated); *10 mM Ca*^*2+*^: 26.5% ± 1.8% (in total 643 cells were evaluated); n>4. (**C-D**) Representative semi-native immunoblots of protein extracts of cells grown on media with and without additional 10 mM Ca^2+^ for 36 h (C) and corresponding densitometric quantification (D) of dimeric αSyn species (as indicated by arrows in (C)). Blots were probed with antibodies directed against αSyn and tubulin as loading control, and the combined signal of the dimeric αSyn species was normalized to tubulin. Means ± s.e.m; n = 10. (**E**) Measurement of cytosolic Ca^2+^ levels in cells equipped with a luminescence-based aequorin reporter construct. After monitoring the basal cytosolic Ca^2+^ levels, Ca^2+^ was automatically injected to the indicated final concentrations to assess the rapid cellular response to the external Ca^2+^ addition. As measurements were performed at the time point of shift to galactose media, corresponding to the Ca^2+^ addition setup described in (A-D), αSyn is not yet expressed. Means ± s.e.m; n = 6. (**F**) Measurement of basal cytosolic Ca^2+^ levels in cells expressing αSyn or harboring the vector control and equipped with the aequorin reporter plasmid. 10 mM Ca^2+^ was added to the culture at the time point of galactose-induced expression and measurements were performed at indicated time points. Means ± s.e.m; n = 4. (**G**) TXRF-based quantification of total cellular Ca^2+^ levels in cells expressing αSyn for 12 h and 24 h supplemented or not with 10 mM Ca^2+^ at the time point of shift to galactose media. Values were normalized to untreated vector control cells at respective time points. Means ± s.e.m; n = 4. (**H**) Determination of calcineurin activity via flow cytometric quantification of GFP intensities in living (PI-negative) cells expressing destabilized GFP (GFP^PEST^) under the control of a calcineurin response element (CDRE). Cells expressing αSyn or harboring the vector control were grown in media with or without 10 mM CaCl_2_. Values have been normalized to t0 (prior to induction of αSyn expression). Means ± s.e.m; n = 12. (**I**) Transient [Ca^2+^]_cyt_ responses measured as described in (E), but 10 mM Ca^2+^ was added to the culture 12 h after shift to galactose media for αSyn promoter induction. Means ± s.e.m; n = 6. (**J**) Determination of calcineurin activity as described in (H), but 10 mM Ca^2+^ was added to the culture 12 h after shift to galactose media for αSyn promoter induction. Values are depicted as fold of t0 as shown in (H). Means ± s.e.m; n = 12. (**K**) Flow cytometric quantification of cell death via PI-staining of cells expressing αSyn or harboring the vector control upon late addition of Ca^2+^. Cells grown on galactose media were analyzed prior to the addition of 10 mM Ca^2+^ (12 h) and at 24 h and 36 h. Means ± s.e.m; n = 4. *p<0.05, **p<0.01, and ***p<0.001.

As we found Ca^2+^-mediated cytoprotection to be dosage-dependent (Fig 1E), we tested whether a further increase of the external Ca^2+^ concentration would prevent toxicity even more efficiently. We added 10 mM or 50 mM Ca^2+^ to the culture media (normal concentration 1 mM) at the time point of galactose-driven induction of αSyn expression and assessed cell death at different time points after the diauxic shift (Fig 2A). Both concentrations delayed cell death with the same efficiency, indicating that maximal cytoprotective effects are already achieved (Fig 2A). Monitoring cell death throughout exponential growth and entry into stationary phase revealed that Ca^2+^ addition already efficiently counteracted the mild αSyn cytotoxicity visible in actively dividing cells (S1 Fig). As Ca^2+^ has been shown to influence the oligomerization and aggregation propensity of αSyn [21,24,53,54], we monitored αSyn-GFP aggregation upon exposure to Ca^2+^ and growth into stationary phase. Confocal microscopy revealed no change of the percentage of cells with large cytosolic αSyn aggregates (Fig 2B). However, semi-native immunoblotting demonstrated that Ca^2+^ administration resulted in decreased abundance of αSyn dimeric species (Fig 2C and 2D). As an increase in cytosolic Ca^2+^ has been shown to promote αSyn oligomerization, likely via direct binding of Ca^2+^ to the C-terminal part of αSyn [21], we next assessed the effect of external Ca^2+^ supplementation on cytosolic Ca^2+^ levels ([Ca^2+^]_cyt_) using yeast cells equipped with the Ca^2+^-dependent luminescent reporter protein aequorin. We observed a rapid and transient peak in [Ca^2+^]_cyt_ as immediate cellular response to the addition of 10 mM and 50 mM Ca^2+^ at the time point of shift to galactose-media and thus prior to αSyn expression (Fig 2E). Cells rapidly restored basal [Ca^2+^]_cyt_, indicating efficient removal from the cytosol. As the time point of Ca^2+^ addition corresponded to the induction of αSyn expression, no effect of αSyn on [Ca^2+^]_cyt_ was visible yet (Fig 2E). Following the basal [Ca^2+^]_cyt_ throughout cellular growth into stationary phase, we found that the rapid and transient cytosolic Ca^2+^ spike seconds after Ca^2+^ addition had hardly any lasting effects on the resting [Ca^2+^]_cyt_ in control cells (Fig 2F). As previously described [26], αSyn caused a progressive elevation of basal [Ca^2+^]_cyt,_ with a maximal amplitude in mid-exponential phase. Notably, the early administration of extra Ca^2+^, concomitant with the induction of αSyn expression, partially correct these αSyn-driven consequences, diminishing the αSyn-induced rise in [Ca^2+^]_cyt_ at all time points (Fig 2F). This drop in [Ca^2+^]_cyt_ was likely due to enforced sequestration of cytosolic Ca^2+^ into organellar stores, as TXRF-based metal analysis revealed a further increase of total cellular Ca^2+^ content (Fig 2G).

The addition of extracellular Ca^2+^ and the subsequent rapid and transient rise in [Ca^2+^]_cyt_ are known to activate calmodulin and in consequence its target calcineurin [55], a Ca^2+^-dependent protein phosphatase closely linked to neurodegeneration. Thus, we monitored calcineurin activity using the calcineurin-dependent response element (CDRE)-driven expression of GFP fused to a PEST-motif, marking it for rapid proteasomal degradation to circumvent accumulation. This setup allows the flow cytometric evaluation of calcineurin dynamics *in vivo* in unperturbed cells and the simultaneous exclusion of confounding dead cells via PI co-staining [56]. As expected, calcineurin activity mirrored [Ca^2+^]_cyt_ kinetics (Fig 2H). Shortly after the external Ca^2+^ pulse, the immediate transient [Ca^2+^]_cyt_ peak translated into a temporary increase in calcineurin activity, visible 1 h after Ca^2+^ addition (inset in Fig 2H). No further deviation from baseline calcineurin activity was detectable in control cells without αSyn expression throughout growth into stationary phase (Fig 2H). However, expression of αSyn resulted in a progressive activation of calcineurin, congruent with the elevation of [Ca^2+^]_cyt_ (Fig 2F). Whereas the early administration of external Ca^2+^ confined the αSyn-induced rise in [Ca^2+^]_cyt_, this did not result in reduced calcineurin activation, indicating that already a persisting 2–3 fold increase of [Ca^2+^]_cyt_ is sufficient to achieve maximal calcineurin activation (Fig 2F–2H). Once cells entered stationary phase, calcineurin activity decreased, concomitant with the drop in [Ca^2+^]_cyt_ (Fig 2H).

Our findings suggest that an early external Ca^2+^ pulse, prior to the onset of αSyn-induced cellular stress, supports cellular Ca^2+^ handling to better cope with the toxic consequences of αSyn expression. To test whether the addition of Ca^2+^ to cells already expressing high levels of αSyn would still efficiently provide cytoprotection, we added 10 mM of Ca^2+^ after 12 h of αSyn expression, the time point of maximal [Ca^2+^]_cyt_ amplitude. This still resulted in a rapid and transient spike of [Ca^2+^]_cyt_ (Fig 2I) but had no prominent effect on calcineurin activity, which was already strongly increased due to αSyn expression (Fig 2J). Notably, this late external Ca^2+^ pulse still efficiently prevented cytotoxicity (Fig 2K). Collectively, these data indicate that the administration of external Ca^2+^, either prior to or during the expression of αSyn, triggers a cellular response that efficiently improves the cell’s capacity to cope with high levels of αSyn.

### Cytoprotection achieved by Ca^2+^ administration requires functional calcineurin signaling

**Fig 3:**
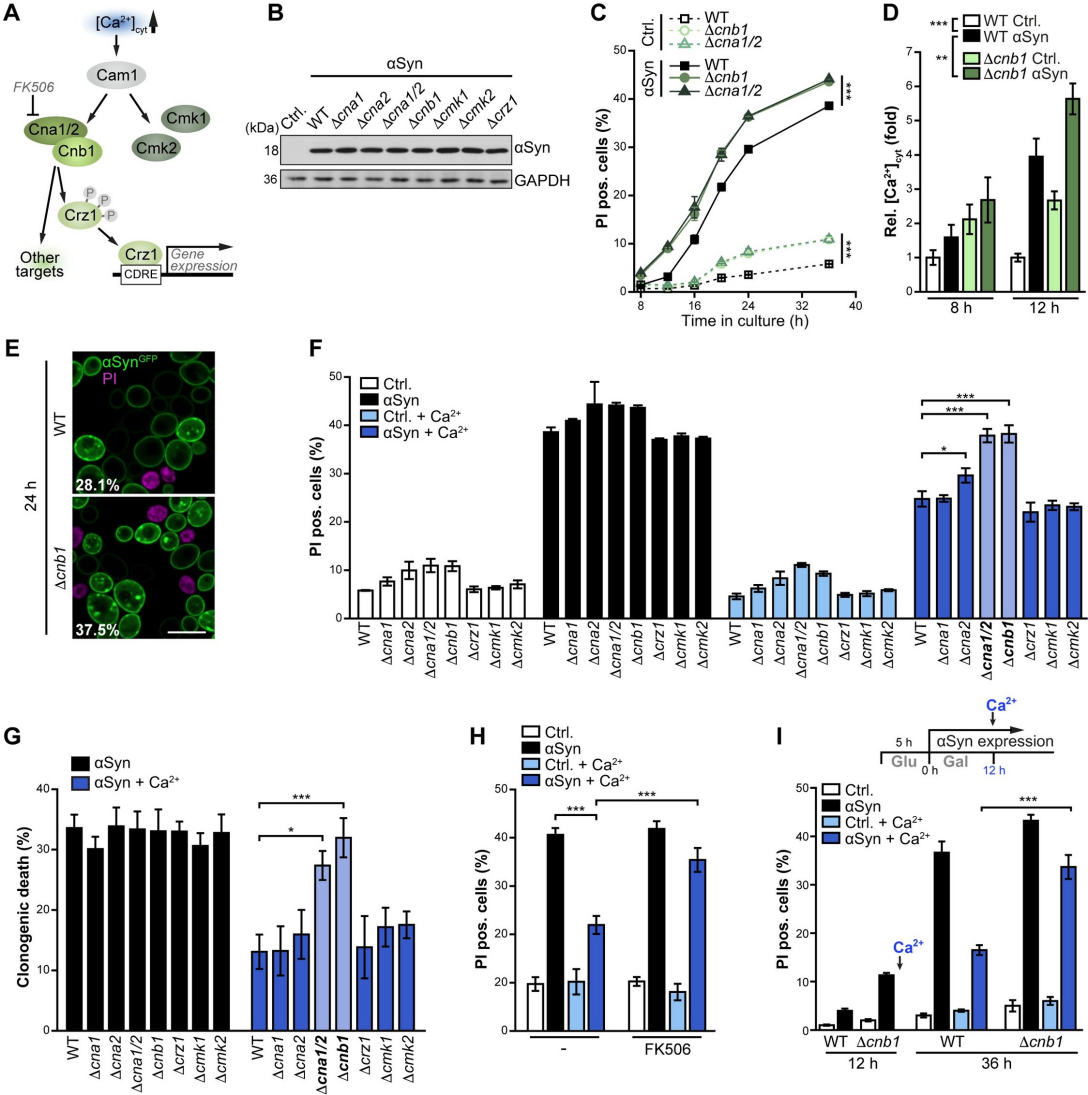
Cytoprotection achieved by Ca^2+^ administration requires functional calcineurin signaling. (**A**) Schematic of the main Ca^2+^ signaling pathway in yeast. Cytosolic Ca^2+^ binds to Calmodulin, which activates the Ca^2+^/calmodulin-dependent protein kinases Cmk1 and Cmk2 and protein phosphatase calcineurin. Calcineurin consists of a regulatory (Cnb1) and one of two catalytic subunits (Cna1 or Cna2) and dephosphorylates numerous cellular targets, among them the calcineurin-responsive zinc finger transcription factor Crz1, which in response translocates into the nucleus to adapt gene expression. Calcineurin can be inhibited by the immunosuppressant drug FK506. (**B**) Immunoblot analysis of protein extracts from WT cells and indicated deletion mutants after 24 h of αSyn expression. Blots were probed with antibodies directed against FLAG-epitope to detect FLAG-tagged αSyn and against glyceraldehyde 3-phosphate dehydrogenase (GAPDH) as a loading control. (**C**) Cell death determined by propidium iodide (PI) staining of WT cells and calcineurin deletion mutants expressing αSyn or harboring the vector control (Ctrl.). Means ± s.e.m; n = 4. (**D**) Aequorin luminescence-based determination of basal cytosolic Ca^2+^ levels in WT and Δ*cnb1* cells expressing *α*Syn for 8 h and 12 h. Data were normalized to WT vector control. Means ± s.e.m; n = 12. (**E**) Confocal micrograph of WT and Δ*cnb1* cells expressing αSyn^GFP^ for 24 h. Cells have been counterstained with PI to visualize dead cells. Scale bar represents 5 μm. Values indicate mean percentages of cells with αSyn^GFP^ aggregates, with the following s.e.m.: WT: 28.1% ± 1.5% (in total 395 cells were evaluated); Δ*cnb1*: 37.5% ± 3.2% (in total 299 cells were evaluated); n≥4. *p<0.05. (**F**) Cell death determined by PI staining of WT cells and indicated deletion mutants expressing αSyn for 36 h or harboring the vector control. Cells were supplemented or not with 10 mM Ca^2+^ at the time point of shift to galactose media. Means ± s.e.m; n = 4. (**G**) Clonogenic death of cells described in (F) grown for 36 h on galactose prior to determination of colony forming units on glucose full media agar plates. Death induced by αSyn was calculated via normalization to isogenic and similarly treated vector control. Means ± s.e.m; n = 8–16. (**H**) Flow cytometric quantification of cell death via PI staining of WT cells expressing αSyn for 36 h or harboring the vector control upon addition of 10 mM Ca^2+^ and 5 μM FK506. Means ± s.e.m; n = 8–12. (**I**) Flow cytometric quantification of cell death via PI staining of WT and Δ*cnb1* cells expressing *α*Syn or harboring the empty vector upon late addition of Ca^2+^. Cells grown on galactose media were analyzed prior to the addition of 10 mM Ca^2+^ (12 h) and at 36 h. Means ± s.e.m; n = 4. *p<0.05, and ***p<0.001.

As αSyn resulted in a hyperactivation of calcineurin, we next analyzed the calmodulin/calcineurin system, the major and evolutionary conserved Ca^2+^ signaling pathway, for an involvement in αSyn cytotoxicity and in Ca^2+^-mediated cytoprotection. Within the complex protein network linking Ca^2+^ signaling and compartmentalization to cellular function in general and to diverse brain-specific processes in particular [57], calmodulin acts as a central intracellular receptor for Ca^2+^. Upon Ca^2+^ binding, calmodulin activates a variety of targets, among them the Ca^2+^/calmodulin-dependent protein kinases Cmk1 and Cmk2 and the protein phosphatase calcineurin (Fig 3A). Calcineurin consists of a catalytic (Cna1 or Cna2) and a regulatory (Cnb1) subunit, is activated via binding to the Ca^2+^/calmodulin complex and modulates the activity of an array of target proteins, including the calcineurin-responsive zinc finger transcription factor Crz1 [58,59]. To test whether genetic ablation of components of this Ca^2+^ signaling branch influences αSyn toxicity, we established αSyn expression in respective deletion mutants (Fig 3B) and monitored the kinetics of αSyn-induced cell death. Toxicity of αSyn was neither affected by the absence of the kinases Cmk1 and Cmk2 nor by the lack of either Cna1 or Cna2 alone, the two isoforms of the catalytic subunit of calcineurin (S2A and S2B Fig). Complete inactivation of calcineurin signaling, achieved either via simultaneous deletion of both *CNA1* and *CNA2* or via deletion of *CNB1*, increased αSyn toxicity to some extend but also resulted in slightly increased cell death *per se* (Fig 3C). Still, the absence of functional calcineurin accelerated αSyn-induced toxicity, resulting in sensitivity towards αSyn already at early time points (Fig 3C). Interestingly, the lack of the calcineurin-responsive transcription factor Crz1 had no impact on αSyn cytotoxicity (S2B Fig). The αSyn-driven rise in [Ca^2+^]_cyt_ was further amplified by genetic inactivation of calcineurin (Fig 3D), and confocal microscopy revealed an increase in cells with large cytosolic αSyn aggregates (Fig 3E). In sum, this supports the notion that the absence of functional calcineurin enhances the deposition αSyn into large cytosolic aggregates and slightly expedites αSyn cytotoxicity, but has no major effect on αSyn-induced cell death.

Next, we tested for a causal role of this Ca^2+^ signaling branch in the suppression of αSyn proteotoxicity via Ca^2+^ supplementation. Inactivation of calcineurin signaling in Δ*cna1/2* and Δ*cnb1* cells inhibited Ca^2+^-mediated cytoprotection as determined via flow cytometric quantification of PI staining, while the administration of Ca^2+^ still efficiently reduced αSyn-induced cell death in all other mutants tested (Fig 3F). Determination of clonogenic death (i.e. the loss of colony forming capacity) confirmed an essential role for calcineurin in Ca^2+^-mediated cytoprotection (Fig 3G). This again was independent of the stress-induced transcriptional reprogramming via the calcineurin-dependent activation of Crz1, as the lack of this major transcription factor had no effect (Fig 3F and 3G). Pharmacological inhibition of calcineurin via the immunosuppressant drug FK506 further corroborated our findings: αSyn proteotoxicity could no longer be prevented by external administration of Ca^2+^ in a scenario where simultaneous addition of FK506 blocked calcineurin activity (Fig 3H). Moreover, the lack of Cnb1 diminished the efficiency of Ca^2+^-induced reduction of αSyn dimeric species (S2C and S2D Fig). Lastly, we also tested whether rescue via late Ca^2+^ addition to cells that already exhibit disrupted Ca^2+^ homeostasis due to prolonged αSyn expression would also require calcineurin. Again, functional calcineurin signaling was crucial for efficient cytoprotection (Fig 3I).

In sum, αSyn disrupts cellular Ca^2+^ handling and triggers a prominent increase in [Ca^2+^]_cyt_ and a concurrent activation of calcineurin signaling. While the inactivation of calcineurin had only minor effects on αSyn proteotoxicity *per se*, it precluded cytoprotection achieved by Ca^2+^ supplementation. Crz1, which coordinates calcineurin-dependent transcriptional reprogramming, was not involved, suggesting that alternative calcineurin targets might contribute to Ca^2+^ -mediated protection.

### Organellar Ca^2+^-sequestration differentially contributes to Ca^2+^-mediated cytoprotection

**Fig 4:**
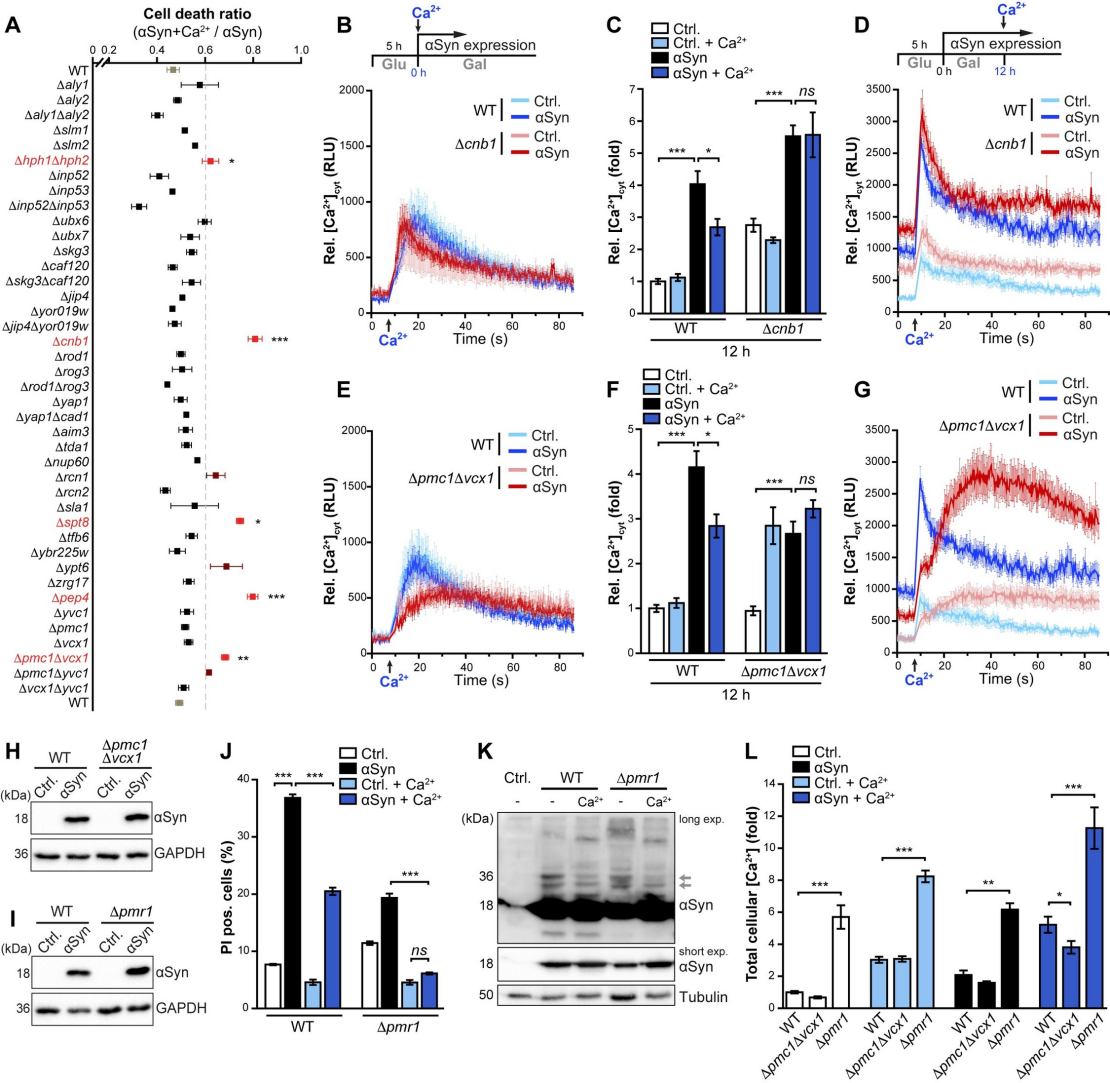
Organellar Ca^2+^-sequestration differentially contributes to Ca^2+^-mediated cytoprotection. (**A**) Cell death determined by propidium iodide (PI) staining of WT cells and indicated deletion mutants, expressing αSyn for 36 h. Untreated cells were compared to cells treated with 10 mM Ca^2+^, and the cell death ratio (αSyn+Ca^2+^ / αSyn) was plotted. Strains above a threshold of 0.6 are depicted in bright red (if different to WT ratio with p<0.05) or in dark red (if not significant). Means ± s.e.m; n = 4–8. (**B-G**) Measurement of basal cytosolic Ca^2+^ levels in WT cells and Δcnb1 mutants (B-D) or Δ*pmc1*Δ*vcx1* mutants (E-G) equipped with the aequorin reporter plasmid and expressing αSyn or harboring the vector control (Ctrl.). Rapid transient [Ca^2+^]_cyt_ responses within 80 s upon early (B, E) or late (D, G) addition of 10 mM Ca^2+^ to cells expressing αSyn for 0 h (B, E) or 12 h (D, G) were quantified. In addition, resting cytosolic Ca^2+^ levels (C, F) were determined 12 h after cells expressing αSyn have been treated with 10 mM Ca^2+^. Means ± s.e.m; n = 6–8. (**H, I**) Immunoblot analysis of protein extracts from WT, Δ*pmc1*Δvcx1 and Δ*pmr1* cells expressing αSyn for 24 h or harboring the vector control. Blots were probed with antibodies directed against FLAG-tagged αSyn and against glyceraldehyde 3-phosphate dehydrogenase (GAPDH) as a loading control. (**J**) Cell death determined by PI staining of WT and Δ*pmr1* cells expressing αSyn for 36 h or harboring the vector control. Cells were grown for 36 h in galactose media with or without the addition of 10 mM Ca^2+^ at the time point of shift to galactose media. Means ± s.e.m; n = 4. (**K**) Representative semi-native immunoblot of protein extracts of WT and Δ*pmr1* cells grown on media with and without additional 10 mM Ca^2+^ for 36 h. Blots were probed with antibodies directed against tubulin as loading control and against αSyn (short and long exposure is shown), and dimeric αSyn species are indicated by arrows. (**L**) TXRF-based quantification of total cellular Ca^2+^ content measured in WT cells and indicated deletion mutants expressing αSyn or harboring the vector control. Cells were grown for 24 h in galactose media with or without addition of 10 mM Ca^2+^ at the time point of shift to galactose media. Data was normalized to untreated WT control. Means ± s.e.m; n = 4. *p<0.05, **p<0.01, and ***p<0.001.

To further identify molecular determinants and processes involved in the cellular response to Ca^2+^ supplementation and the mitigation of αSyn proteotoxicity, we screened 41 yeast deletion mutants connected to calcineurin signaling and Ca^2+^ homeostasis (Fig 4A). This included mutants lacking previously identified direct substrates of calcineurin, among them proteins involved in membrane structure and function, ubiquitin signaling, transcription and translation, protein transport and Ca^2+^ signaling [60]. Moreover, as the vacuole serves as main Ca^2+^ reservoir in yeast, we additionally assessed mutants devoid of proteins involved in vacuolar Ca^2+^ transport and function. In most strains, the inhibition of αSyn-induced cell death by Ca^2+^ administration was efficiently maintained (Fig 4A). However, the beneficial effects of Ca^2+^ were compromised in cells lacking the calcineurin targets Hph1 and Hph2, two homologous ER-proteins involved in stress response and necessary for proper biogenesis and assembly of the vacuolar H^+^-ATPase governing vacuolar acidification [61]. Furthermore, depleting vacuolar Ca^2+^ via simultaneous inactivation of the two main vacuolar Ca^2+^ transporters Pmc1 and Vcx1 as well as lack of Pep4, the yeast orthologue of mammalian Cathepsin D (CatD) and major vacuolar protease, hampered Ca^2+^-mediated cytoprotection. In addition, Spt8, a subunit of the Spt-Ada-Gcn5 acetyltransferase (SAGA) complex and a calcineurin substrate, was required for Ca^2+^-mediated cytoprotection. Interestingly, the SAGA subunit Spt7 has been shown to be cleaved by Pep4, which results in the loss of Spt8 from the SAGA complex and the formation of a SAGA-like (SLIK) complex [62,63]. Altogether, these data clearly point to a crucial role of vacuolar function in Ca^2+^-mediated mitigation of αSyn proteotoxicity.

To further assess the contribution of cellular and in particular vacuolar Ca^2+^ handling to αSyn proteotoxicity and Ca^2+^-mediated cytoprotection, we analyzed cytosolic Ca^2+^ levels in Δ*cnb1* (Fig 4B–4D) and in Δ*pmc1*Δ*vcx1* mutants (Fig 4E–4G). We monitored (i) the rapid peak of [Ca^2+^]_cyt_ immediately after early Ca^2+^ addition, (ii) basal [Ca^2+^]_cyt_ after prolonged αSyn expression and Ca^2+^ treatment for 12 h, and (iii) the rapid [Ca^2+^]_cyt_ response upon late Ca^2+^ addition. The loss of calcineurin signaling had no prominent effect on the rapid, transient [Ca^2+^]_cyt_ peak immediately after the early or late Ca^2+^ addition, and restoration of resting [Ca^2+^]_cyt_ was quite comparable to wild type cells (Fig 4B–4D). However, Ca^2+^ administration no longer mitigated the increase of basal [Ca^2+^]_cyt_ driven by αSyn in absence of functional calcineurin (Fig 4C). In cells lacking Pmc1 and Vcx1, [Ca^2+^]_cyt_ levels were strongly elevated in particular upon late addition of Ca^2+^ to cells expressing αSyn (Fig 4G), likely because the removal of Ca^2+^ from the cytosol into the vacuole upon supplementation of Ca^2+^ was compromised. Overall expression levels of αSyn were not affected (Fig 4H). In addition to the vacuole, the ER and Golgi represent important lumenal stores for cellular Ca^2+^. Transport of Ca^2+^ into these secretory compartments mainly depends on the action of the phylogenetically conserved Ca^2+^/Mn^2+^ ATPase Pmr1, the yeast ortholog of mammalian SPCA. The loss of Pmr1 results in decreased Ca^2+^ in the ER and Golgi while increasing vacuolar Ca^2+^ sequestration [64,65] and suppresses αSyn toxicity in several PD model systems [26]. To test whether loss of Ca^2+^ sequestration into the ER and Golgi in absence of Pmr1 would affect Ca^2+^-mediated cytoprotection, we expressed αSyn in Δ*pmr1* cells (Fig 4I) and administered extra Ca^2+^. Notably, Ca^2+^ addition completely prevented αSyn-induced cell death in cells lacking Pmr1, restoring cellular viability back to wild type control cells (Fig 4J). Semi-native immunoblotting demonstrated a decrease in αSyn dimeric species in Δ*pmr1* cells and a slight accumulation of high-molecular weight species. Supplementation with Ca^2+^ resulted in the disappearance of these larger species and further reduced dimeric αSyn species (Fig 4K). TXRF-based metal analysis revealed that Ca^2+^-mediated cytoprotection and the change in αSyn oligomerization was accompanied by a massive increase of total cellular Ca^2+^ (Fig 4L). As sequestration into the secretory pathway is compromised in cells devoid of Pmr1, this most probably depicts increased Ca^2+^ storage within the vacuole. In line, the boost of total cellular Ca^2+^ load upon Ca^2+^ administration was reduced in cells deficient in vacuolar Ca^2+^ sequestration due to lack of Pmc1 and Vcx1 (Fig 4L). This supports the notion that increasing vacuolar Ca^2+^ storage capacity is beneficial to maintain cellular fitness despite high levels of αSyn.

In aggregate, these findings suggest that an external Ca^2+^ pulse triggers a cellular response that mitigates the αSyn-driven rise in [Ca^2+^]_cyt_ in a calcineurin-dependent way and leads to the sequestration of excess Ca^2+^ into the vacuole via cooperative action of the vacuolar transporters Pmc1 and Vcx1. Moreover, combining genetic inactivation of Pmr1, which prevents sequestration of Ca^2+^ into the secretory pathway and potentiates its transport into the vacuole, with external Ca^2+^ addition reduces αSyn oligomerization and completely prevents αSyn proteotoxicity.

### Ca^2+^-addition triggers calcineurin-dependent hyperactivation of Pep4

**Fig 5:**
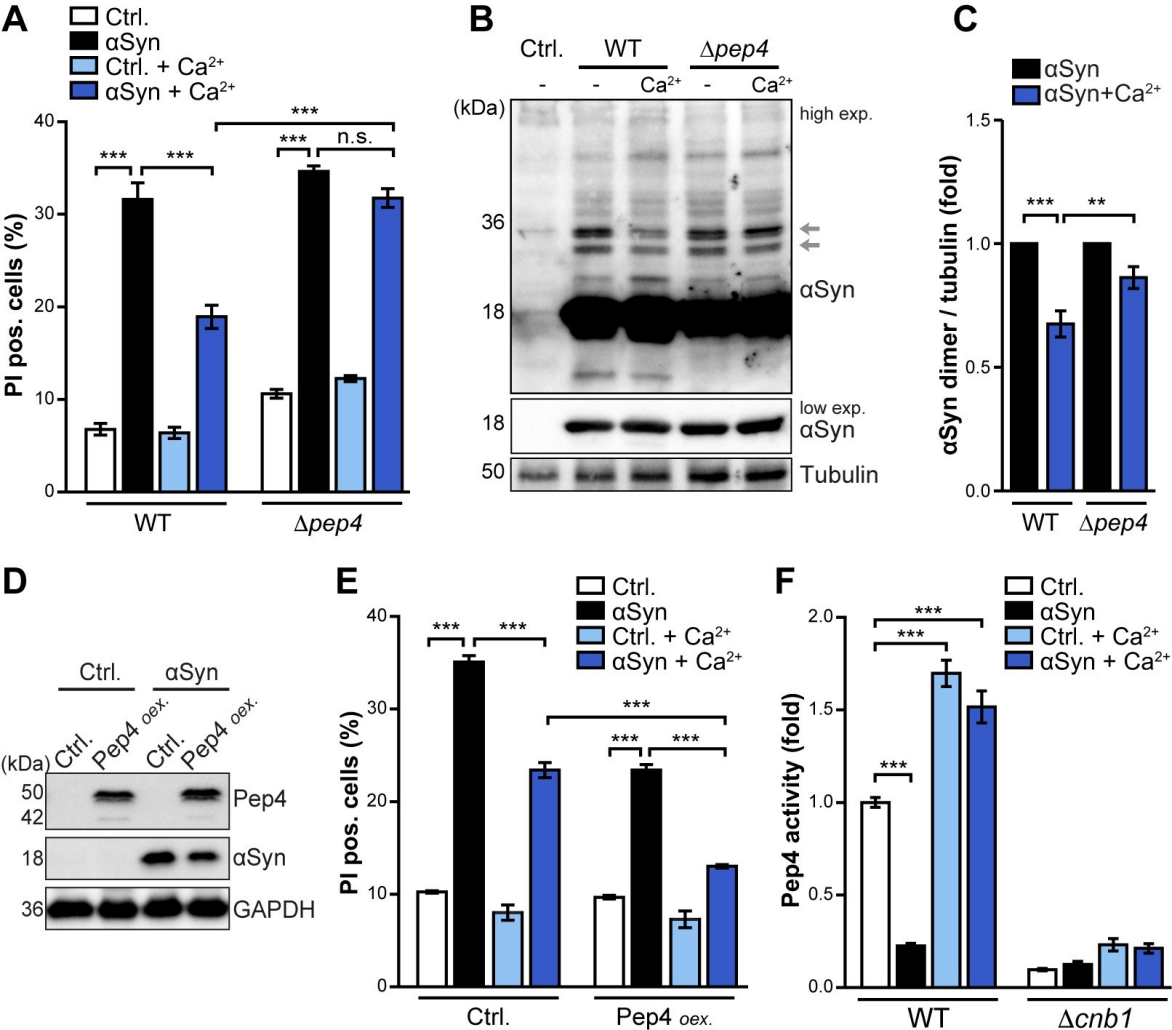
Ca^2+^-addition triggers calcineurin-dependent hyperactivation of Pep4. (**A**) Cell death determined by propidium iodide (PI) staining of WT and Δ*pep4* cells, expressing αSyn for 36 h or harboring the vector control (Ctrl.) with or without addition of 10 mM Ca^2+^. Means ± s.e.m; n = 4. (**B, C**) Representative semi-native immunoblots of protein extracts of cells grown on media with and without additional 10 mM Ca^2+^ for 36 h (B) and corresponding densitometric quantification (C) of dimeric αSyn species (as indicated by arrows in (B)). Blots were probed with antibodies directed against αSyn and tubulin as loading control, and the combined signal of the dimeric αSyn species was normalized to tubulin. Means ± s.e.m; n = 5. (**D**) Immunoblot analysis of protein extracts from cells expressing αSyn and additionally overexpressing Pep4 (Pep4 *oex*.) under the control of a galactose promoter. Blots were probed with antibodies against FLAG-tagged αSyn and Pep4, as well as GAPDH as loading control. (**E**) Cell death determined by PI staining of cells described in (D). Cells were treated with 10 mM Ca^2+^ at the time point of induction of galactose-driven expression of αSyn and Pep4 (Pep4 *oex*.). Means ± s.e.m; n = 4. (**F**) Biochemical measurement of Pep4 proteolytic activity in WT or Δ*cnb1* cells expressing αSyn for 16 h or harboring the vector control with or without addition of 10 mM Ca^2+^. Means ± s.e.m; n = 4. *p<0.05, **p<0.01, ***p<0.001, and *n*.*s*. not significant.

Besides calcineurin signaling and vacuolar Ca^2+^ transport, we also identified the vacuolar protease Pep4, yeast CatD, as crucial for Ca^2+^-mediated cytoprotection. The lack of Pep4 completely precluded the beneficial effects of Ca^2+^ addition on cellular survival (Fig 5A). Moreover, functional Pep4 was required for efficient Ca^2+^-induced reduction of αSyn dimeric species (Fig 5B and 5C). *Vice versa*, overexpression of Pep4 decreased αSyn protein abundance and reduced αSyn proteotoxicity (Fig 5D and 5E), in line with our previous results [66]. A combination of high levels of Pep4 and simultaneous Ca^2+^ supplementation inhibited cell death even more efficiently, arguing for additive cytoprotection conferred by these two interventions (Fig 5E). Biochemical quantification of Pep4 activity revealed a prominent reduction of proteolytic capacity upon αSyn expression, which could be restored by Ca^2+^ administration (Fig 5F). Remarkably, the administration of Ca^2+^ not only completely blocked the αSyn-driven reduction of Pep4 activity, but even overcompensated, leading to hyperactivity of this protease in all cells independent of αSyn expression (Fig 5F). As we have recently established a link between calcineurin signaling and efficient trafficking of Pep4 to the vacuole [66], we tested whether calcineurin would be required for the Ca^2+^-mediated boost of vacuolar proteolysis. Indeed, the lack of Cnb1 not only reduced Pep4 activity *per se*, but also completely prevented the activation of Pep4 by Ca^2+^ administration (Fig 5F). Thus, Ca^2+^-administration triggers a calcineurin-dependent hyperactivation of Pep4, which in consequence improves survival of cells expressing αSyn.

### Administration of Ca^2+^ activates CatD in Drosophila and prevents αSyn proteotoxicity

**Fig 6:**
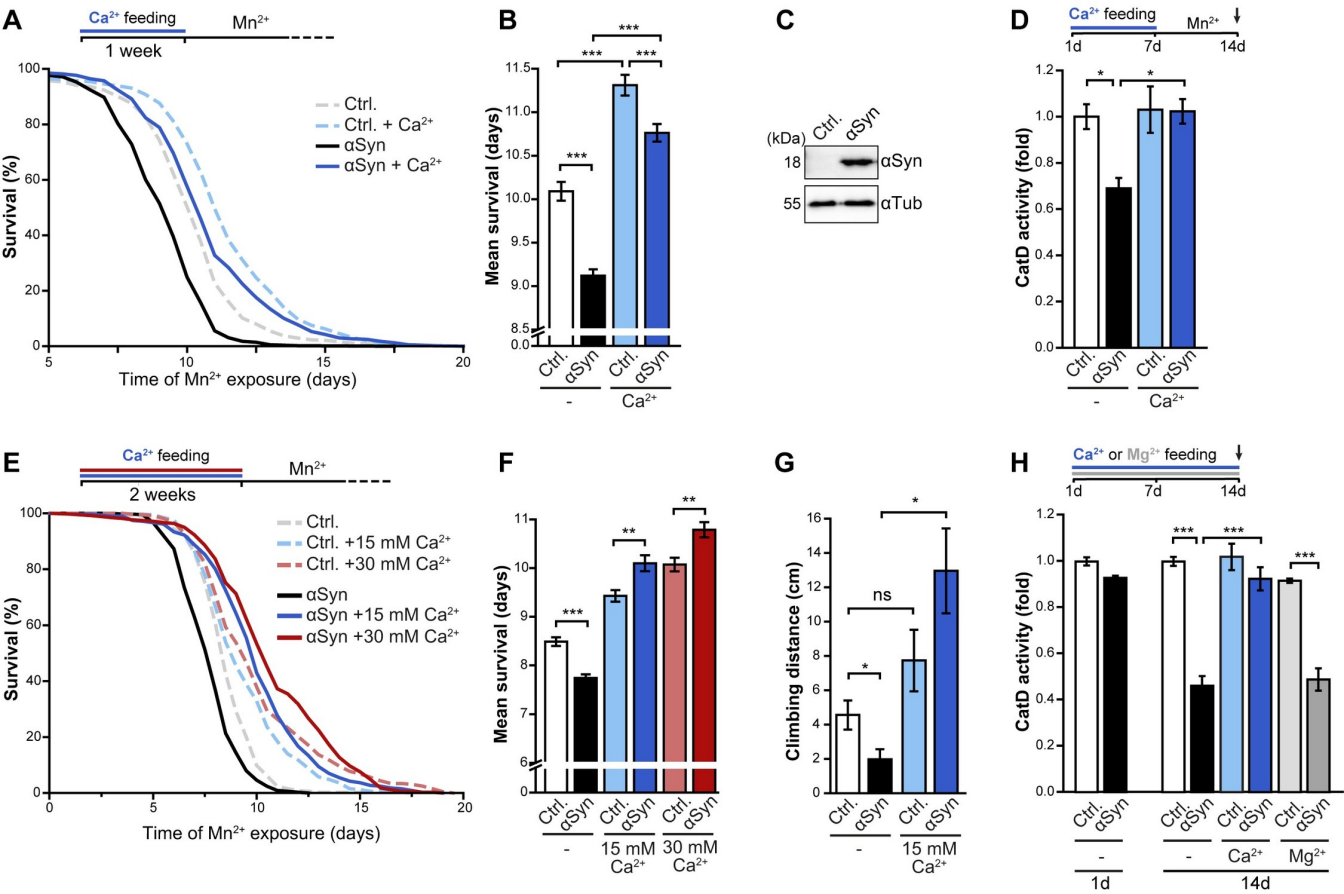
Administration of Ca^2+^ activates Cathepsin D in Drosophila and prevents αSyn proteotoxicity. (**A-B**) Survival of flies neuronally expressing human αSyn (UAS-αSyn) driven by *nsyB-Gal4* and corresponding control flies upon exposure to 10 mM Mn^2+^. 1–3 days old female flies were collected and kept on food with or without additional 15 mM Ca^2+^ for 1 week prior to Mn^2+^ exposure. Kaplan-Meier survival analysis (A) and corresponding mean survival (B) is shown. In total, 470–560 flies per genotype and condition were evaluated, and 30 flies were kept per vial. (Genotypes: Ctrl. = Nsyb-Gal4>w^1118^; αSyn = Nsyb-Gal4>UAS-αSyn). (**C**) Immunoblot analysis of head lysates from flies described in (A). Blots were probed with antibodies directed against αSyn and tubulin (αTub) as loading control. (**D**) Biochemical measurement of cathepsin D (CatD) proteolytic activity in head lysates of flies described in (A). Prior to Mn^2+^ stress, flies were kept on food with or without additional 15 mM Ca^2+^ for 1 week. Heads were collected after 7 days of Mn^2+^ treatment. Means ± s.e.m; n = 6–7. **(E-F)** Survival of flies neuronally expressing αSyn (UAS-αSyn) driven by *nsyB-Gal4* and corresponding control flies upon exposure to 10 mM Mn^2+^. 1–3 days old female flies were collected and kept on food with or without additional 15 mM Ca^2+^ or 30 mM Ca^2+^ for 2 weeks prior to Mn^2+^ exposure. Kaplan-Meier survival analysis (E) and corresponding mean survival (F) is shown. In total, 340–400 flies per genotype and condition were evaluated, and 30 flies were kept per vial. (**G**) Climbing activity of flies described in (E) after 6 days of Mn^2+^ treatment. Means ± s.e.m; n = 6 with 6–8 flies per vial. (**H**) Biochemical measurement of CatD proteolytic activity in head lysates of flies neuronally expressing αSyn (UAS-αSyn) driven by *nsyB-Gal4* and corresponding control flies. Heads were collected at day 1 (on standard food without additional Ca^2+^ or Mg^2+^ supplementation) and after 14 days, during which flies were kept on food supplemented with either 15 mM Ca^2+^, 15 mM Mg^2+^ or without supplementation as indicated. Means ± s.e.m; n = 4. *p<0.05, **p<0.01, ***p<0.001, and *n*.*s*. not significant.

Finally, we tested for phylogenetic conservation of the cytoprotective effects of Ca^2+^ administration. To this end, we used a Drosophila model for PD based on the pan-neuronal expression of human αSyn under the control of the *UAS-GAL4* system. We combined this genetic trigger for PD with manganese treatment, an environmental risk factor for PD [44]. This setup was reported to cause the death of flies within days [26,41]. To increase Ca^2+^ intake, we supplemented the food with additional 15 mM Ca^2+^ for 1 week prior to exposure to manganese. The xenotopic expression of αSyn selectively in the fly brain accelerated manganese toxicity, leading to significantly decreased survival compared to control flies (Fig 6A–6C). However, pre-feeding of these flies with extra Ca^2+^ for 1 week prior to manganese exposure prevented αSyn-driven organismal death. Of note, Ca^2+^ pre-feeding not only precluded αSyn toxicity but also efficiently counteracted the toxic consequences of manganese overload *per se* (Fig 6A and 6B). Next, we explored a role for Drosophila CatD in the pro-survival effect of Ca^2+^ feeding. The neuronal expression of αSyn clearly compromised CatD function, as indicated by biochemical quantification of CatD activity in fly head lysates after 1 week of manganese exposure (Fig 6D). This defect was absent in flies pre-fed with extra 15 mM Ca^2+^ prior to manganese stress (Fig 6D). Remarkably, extending the Ca^2+^ pre-feeding period blocked αSyn toxicity completely. In fact, when keeping flies on food supplemented with extra Ca^2+^ for 2 weeks prior to exposure to manganese, the neuronal expression of αSyn even provided cytoprotection (Fig 6E and 6F). Thus, depending on the environmental availability of Ca^2+^, αSyn either expedites or counteracts manganese-induced neurotoxicity. Similarly, toxic *versus* protective effects of αSyn were apparent when monitoring motor function (Fig 6G). Neuronal expression of αSyn clearly compromised climbing ability, measured as capacity of negative geotaxis, upon exposure to manganese. However, when flies were pre-fed with Ca^2+^ for 2 weeks, αSyn no longer compromised but instead improved motor function (Fig 6G). These data suggest a beneficial function of αSyn in neurons upon manganese overload that is highly dependent on Ca^2+^ availability.

Finally, we tested for an effect of αSyn on CatD activity in absence of manganese as additional toxic trigger and thus conditions where neuronal αSyn expression did not yet affect Drosophila survival. Indeed, in flies aged for 2 weeks on regular food, the neuronal expression of αSyn already prominently reduced CatD proteolysis (Fig 6H). Supplementation of food with 15 mM Ca^2+^ (but not with 15 mM Mg^2+^) completely prevented these pathological changes (Fig 6H). Hence, increasing Ca^2+^ availability precludes αSyn-induced lysosomal dysfunction, efficiently prevents αSyn toxicity upon manganese overload and discloses a cytoprotective function of αSyn within Drosophila neurons.

## Discussion

Defects in the lysosomal pathway are intimately linked to the pathogenesis of neurodegenerative disorders associated with proteotoxic stress, such as PD [36,38,39]. In this study, we establish a novel regime to re-activate lysosomal degradative capacity and provide insights into the underlying molecular circuits. We demonstrate that administration of Ca^2+^ prevents αSyn proteotoxicity in yeast and Drosophila models for PD and link this cytoprotection to the activation of lysosomal CatD proteolysis. While αSyn prominently decreased CatD activity in yeast cells and fly brains, extra Ca^2+^ completely restored this defect, thus efficiently equipping the lysosome for proteolytic breakdown. This compensates for the proteostatic stress posed by high αSyn levels and improves cellular and organismal viability.

**Fig 7:**
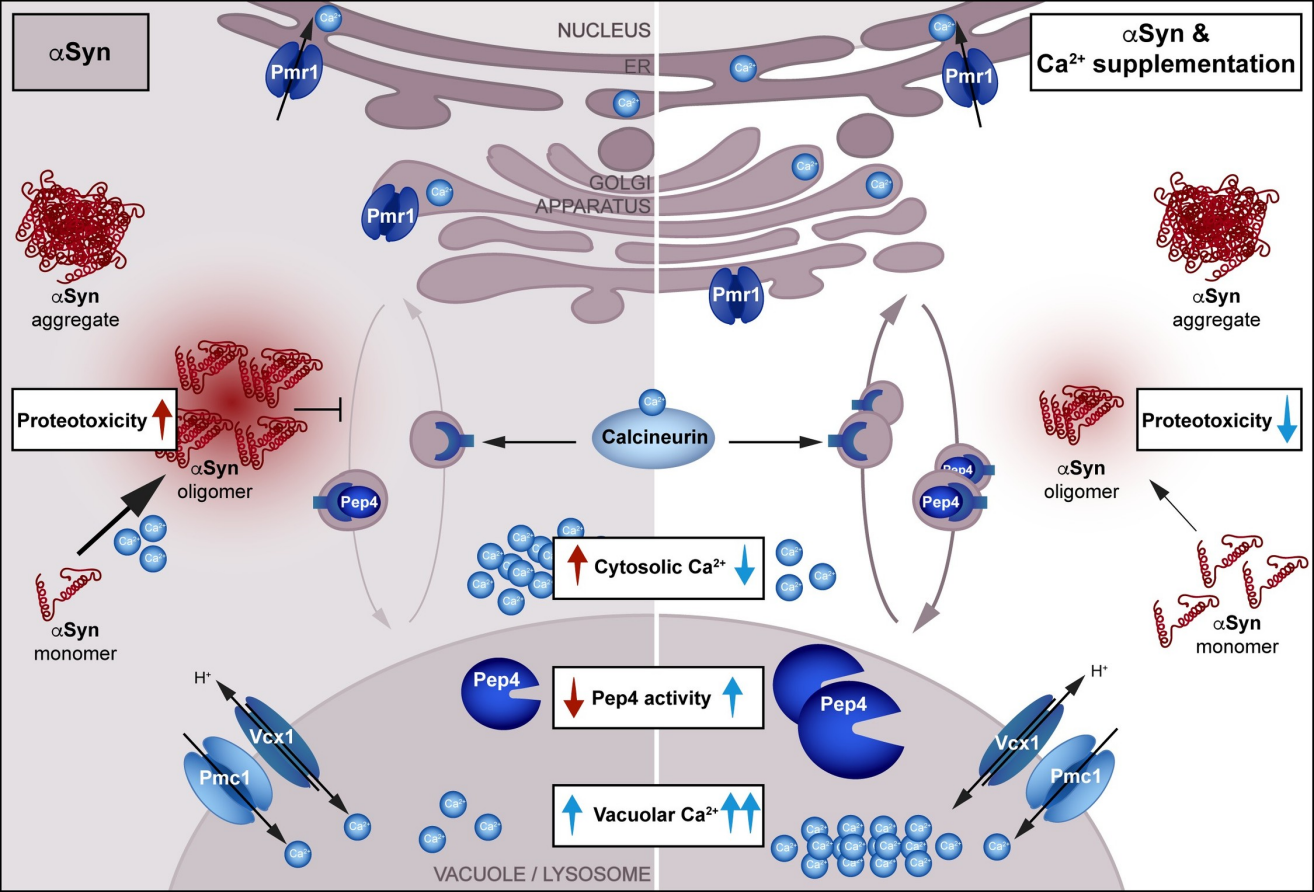
Schematic overview of αSyn proteotoxicity and the calcineurin-dependent stimulation of vacuolar proteolysis upon Ca^2+^ supplementation. Upon heterologous expression of human αSyn, oligomers and large cytosolic aggregates form and cytosolic Ca^2+^ levels increase, which might in turn further accelerate αSyn oligomerization. αSyn impairs Pep4/CatD proteolytic activity, most probably via interference with calcineurin-dependent recycling of the shuttling receptor of Pep4/CatD. In turn, decreased vacuolar/lysosomal proteolytic capacity leads to cell death (left panel). Supplementation with Ca^2+^ reduces αSyn oligomerization, mitigates the αSyn-driven rise in cytosolic Ca^2+^ via sequestration into organellar stores, in particular the vacuole, and stimulates calcineurin-dependent shuttling of Pep4/CatD and thus vacuolar proteolytic capacity. Collectively, this decreases αSyn proteotoxicity and supports viability. Loss of Ca^2+^ transport into the secretory pathway via genetic inactivation of Pmr1 even increases the cytoprotective capacity of Ca^2+^ supplementation.

Conceptually, cytoprotection mediated by Ca^2+^ supplementation seems to involve different, likely interrelated processes. On the one hand, Ca^2+^ addition drives a cellular response that adjusts cell physiology and Ca^2+^ handling to better cope with the toxic effects of αSyn. On the other hand, it affects αSyn itself and alters its oligomerization propensity, thereby mitigating its toxic effects on Ca^2+^ homeostasis and lysosomal function (Fig 7). αSyn causes a massive increase in cytosolic Ca^2+^, which, in turn, can induce αSyn oligomerization [21,22], likely representing a self-amplifying loop. The administration of Ca^2+^ results in a rapid but transient rise of cytosolic Ca^2+^, followed by efficient sequestration into organellar stores to restore resting cytosolic Ca^2+^ levels. The sustained increased availability of Ca^2+^ in the environment provokes an adjustment of cellular Ca^2+^ handling that requires calcineurin signaling and efficient sequestration of cytosolic Ca^2+^ into the vacuole. This, in turn, attenuates the αSyn-induced elevation of resting cytosolic Ca^2+^ levels, which in consequence may be responsible for the reduced abundance of αSyn oligomers, assumed to be the primary toxic αSyn species.

In addition, we find that organellar Ca^2+^ sequestration differentially contributes to Ca^2+^-mediated cytoprotection. In yeast, Ca^2+^ mainly accumulates within the vacuole or is sequestered into the secretory pathway, while mitochondria lack selective Ca^2+^ channels and seem to play a minor role in Ca^2+^ storage [67–69]. Our results suggest that Ca^2+^ sequestration into the vacuole is necessary for efficient Ca^2+^-mediated rescue, while transport into the secretory pathway is dispensable. In fact, inactivation of the secretory pathway Ca^2+^/Mn^2+^ ATPase Pmr1 even supported the beneficial effects of Ca^2+^ administration, resulting in a reduction of αSyn oligomeric species and a complete absence of αSyn proteotoxicity. We have previously shown that cytosolic Ca^2+^ overload and subsequent cellular demise driven by high levels of αSyn requires the Ca^2+^-transporting activity of Pmr1, a pump causal for αSyn toxicity in yeast, nematode and fly models for PD [26]. *Vice versa*, overexpression of Pmr1 massively aggravated cell death induced by αSyn [26]. Supporting the notion that inactivation of Ca^2+^ transport into the secretory pathway alleviates αSyn toxicity, the pharmacological inhibition of the *C*. *elegans* sarco/endoplasmic reticulum Ca^2+^ ATPase SERCA via cyclopiazonic acid prevented αSyn-induced dopaminergic neuron loss. Moreover, treatment with cyclopiazonic acid restored normal cytosolic Ca^2+^ levels in a primary neuronal αSyn model [33]. In line with this, our data indicate that Δ*pmr1* cells, harboring a reduced potential to sequester Ca^2+^ into the secretory pathway but instead strongly accumulating Ca^2+^ in the vacuole [64,65,70], are less susceptible to the toxic effects of αSyn expression. Moreover, these cells are completely resistant to αSyn toxicity when additionally supplied with high external Ca^2+^. Supplementation with Ca^2+^ not only mitigated the αSyn-driven elevation of cytosolic Ca^2+^ but also massively increased total cellular Ca^2+^ content, reflecting uptake into the vacuole as main organellar Ca^2+^ depot. Reminiscent of recent findings demonstrating that increased vacuolar Ca^2+^ sequestration via overexpression of Pmc1 can reduce αSyn-induced growth arrest [27], lack of the vacuolar Ca^2+^ transporters Pmc1 and Vcx1, reported to decrease vacuolar Ca^2+^ storage [64,71], precluded efficient cytoprotection via Ca^2+^ supplementation, suggesting a causal contribution of the vacuole. Moreover, increased vacuolar Ca^2+^ sequestration coincided with a stimulation of CatD activity that strictly required functional calcineurin signaling and was critical for cytoprotection. Administration of Ca^2+^ no longer prevented αSyn proteotoxicity in cells lacking CatD, demonstrating a key role for calcineurin-dependent stimulation of this vacuolar aspartyl protease in Ca^2+^-mediated cytoprotection. In different PD model systems, high levels of αSyn have been shown to impair CatD activity, and, *vice versa*, the overexpression of CatD reduced αSyn toxicity as well as the abundance of αSyn oligomers and aggregates [66,72–74]. Compromised CatD proteolysis was caused by defective function of its sorting receptor, the mannose 6-phosphate receptor, which mislocalized to the lysosomal membrane instead of shuttling between the *trans*-Golgi and the lysosome in successive rounds of transport [66,72]. We have recently demonstrated that functional calcineurin signaling is required for efficient trafficking and recycling of Pep1, the yeast mannose-6-phosphate receptor, and thus delivery of yeast CatD to the vacuole and the cytoprotective effects of CatD overexpression [66]. In line, we now show that genetic inactivation of calcineurin not only compromises CatD activity *per se* but also completely abrogates the boost of CatD activity achieved by Ca^2+^ supplementation. Moreover, efficient Ca^2+^-mediated reduction of αSyn dimeric species involved both calcineurin and CatD. As shown previously, calcineurin is critical for the efficient retrieval of Pep1 from the vacuole back to the *trans*-Golgi network [66], a process mediated by the retromer complex. Malfunction of this highly conserved multimeric complex via point mutation of its component Vps35 has been linked to different forms of PD [75] and results in insufficient CatD activity and lysosomal dysfunction in different PD model systems [76–79]. Still, whether calcineurin contributes to efficient retromer-mediated recycling of Pep1 and thus CatD trafficking remains to be tested. Alternatively, calcineurin might contribute to Ca^2+^ signaling-mediated dephosphorylation of Ykt6, a SNARE with critical roles in CatD trafficking and Golgi membrane fusion implicated in αSyn toxicity [80–82].

The involvement of calcineurin in neurodegeneration seems rather complex, as both excessive and insufficient activity are associated with neuronal dysfunction [83–87]. In several PD models, an intermediate activity of calcineurin prevented αSyn toxicity, while complete loss and hyperactivation amplified proteotoxicity [34]. A direct interaction between αSyn and calcineurin has been demonstrated *in vitro* [88], but whether such an interaction contributes to observed phenotypes is subject to speculation. While the regulatory circuits determining cytocidal *versus* cytoprotective effects remain yet elusive, the fine-tuning of calcineurin activity seems to determine the cellular response to αSyn [34,66]. We find calcineurin to impact on αSyn proteotoxicity and to support Ca^2+^-mediated rescue at different levels. Though the prominent rise of cytosolic Ca^2+^ levels upon αSyn expression translated into a hyperactivation of calcineurin, the absence of calcineurin had only minor effects on αSyn toxicity *per se*, although it increased the abundance of large αSyn aggregates. However, upon Ca^2+^ supplementation, calcineurin was critical to equip the vacuole with CatD and to restore lysosomal proteolysis, likely by supporting the trafficking of CatD via the mannose-6-phosphate receptor Pep1. In addition, calcineurin enabled the cell to efficiently cope with αSyn-induced disturbances of Ca^2+^ homeostasis, thereby diminishing the αSyn-driven elevation of cytosolic Ca^2+^ levels. As such, at least basal activity of calcineurin is required to achieve cytoprotection by Ca^2+^.

Interestingly, in particular dopaminergic neurons seem sensitive to perturbations in Ca^2+^ homeostasis [89–91]. In contrast to the vast majority of neurons in the brain, adult dopaminergic neurons strongly rely on specific voltage-dependent Ca^2+^ channels to drive rhythmic pace-making [90,92]. With progressing age, this causes a sustained increase in cytosolic Ca^2+^ levels, critically contributing to the highly cell-type specific decay of dopaminergic neurons during PD [90,93]. Moreover, voltage-gated Ca^2+^ entry seems intimately connected to PD pathology in general and αSyn toxicity in particular [21,35,94,95]. Still, despite clear evidence that high cytosolic Ca^2+^ and αSyn in combination drive PD-associated neuronal degeneration, the voltage-gated Ca^2+^ channel blocker isradipine did not slow the progression of early-stage PD in clinical trials [96], and observational studies in respect to serum Ca^2+^ levels in PD patients are inconclusive [97].

Adding to a highly complex connection between Ca^2+^ homeostasis and αSyn, cellular Ca^2+^ handling does not only impact the toxicity of αSyn but also its physiological role in vesicle clustering in the pre-synaptic terminal [21]. In line with Ca^2+^ as a critical regulator of the physiological function of αSyn in neurons, we demonstrate that Ca^2+^ bioavailability determines toxic *versus* beneficial effects of αSyn when xenotopically expressed in Drosophila neurons. While pan-neuronal expression of αSyn resulted in increased death of flies exposed to manganese, pre-feeding with extra Ca^2+^ prior to manganese treatment not only prevented toxicity but disclosed a neuroprotective function of αSyn. When kept on food supplemented with extra Ca^2+^, neuronal expression of αSyn prevented manganese-induced motor dysfunction and extended Drosophila survival. Interestingly, in neuronal cell culture, physiological levels of αSyn have already been suggested to prevent manganese-induced neurotoxicity [98], and lack of αSyn aggravated motor deficits in mice exposed to manganese [99]. In addition, expression of αSyn has been suggested to prevent acute manganese toxicity in *C*. *elegans* devoid of orthologs of additional PD-associated genes implicated in oxidative stress pathways [100]. Thus, depending on the respective genetic setup, αSyn can either enhance or reduce manganese-induced cellular decline, and this seems highly depended on Ca^2+^ availability.

Collectively, our results suggest an evolutionary conserved mechanism by which early and late Ca^2+^ administration adjusts cellular Ca^2+^ handling and stimulates lysosomal proteolytic activity in a calcineurin-dependent manner, resulting in a reduction of αSyn proteotoxicity. Whether this will be transferable to other neurotoxic proteins remains to be investigated. However, in light of the highly conserved fundamental processes compromised by αSyn and corrected by Ca^2+^ administration, this regime may improve the cell’s capacity to cope with proteotoxic stress in general.

## Material and methods

### *S*. *cerevisiae* strains and genetics

Experiments were carried out in BY4741 (MATa *his3Δ1 leu2Δ0 met15Δ0 ura3Δ0*) and respective null mutants. Notably, phenotypes observed in null mutants obtained from Euroscarf were confirmed with handmade deletion mutants. All strains used in this study are listed in S1 Table. Yeast plasmid transformation was performed using a standard lithium acetate method [101]. Previously described αSyn-constructs in pESC-His and pESC-Ura (Stratagene) were used, coding for C-terminally FLAG-tagged (in pESC-his) or C-terminally GFP-tagged (in pESC-Ura) versions of human αSyn under the control of a GAL10 promoter [41,66,102]. For co-expression of αSyn and Pep4, recently described constructs in pESC-Ura for αSyn and pESC-His for Pep4 were used [66]. Generation of deletion mutants of interest was performed according to Janke et al. using the pFA6a-hphNTI plasmid to generate knockout cassettes containing a hygromycin B selection marker [103]. All primers used are listed in S2 Table.

### Yeast culture conditions and analysis of viability

All strains were grown on synthetic complete (SC) medium containing 0.17% yeast nitrogen base (Difco), 0.5% (NH_4_)_2_SO_4_ and 30 mg/l of all amino acids (except 80 mg/l histidine and 200 mg/l leucine), 30 mg/l adenine, and 320 mg/l uracil with either 2% glucose (SCD) or 2% galactose (SCG) for induction of GAL10-driven expression of αSyn. To determine growth, survival, oxidative stress and loss of membrane integrity, cells from SCD overnight cultures were inoculated in fresh SCD to OD_600_ 0.1, grown to midlog phase (OD_600_ 0.3–0.35) and shifted to SCG for induction of αSyn expression. Immediately after the shift to SCG, cells were treated with indicated concentrations of metals using stocks of 1 M NaCl, 1 M Mg_2_SO_4_, 1 M KCl, 1 M CaCl_2_, 1 M MnCl_2_, 0.5 M Fe_2_SO_4_, 1 M Cu(NO_3_)_2_ and 1 M ZnCl_2_ in ddH_2_O. For ‘late addition’ of Ca^2+^, cells were treated with 10 mM CaCl_2_ at 12 h after shift to SCG. For pharmacological inhibition of calcineurin activity, media was supplemented with 5 μM FK506 (Sigma; stock 2.5 mM in DMSO). For clonogenic survival plating, aliquots were taken at indicated time points and colony-forming units (CFU) were determined as previously described [26,104]. Briefly, a CASY cell counter (Schärfe systems) was used to measure the cell counts, 500 cells were plated on full media (YEPD) agar plates and CFU were quantified after two days of growth using a Scan300 (Interscience). To measure loss of membrane integrity, cultures were subjected to propidium iodide (PI)-staining after indicated time of αSyn expression. To this end, about 2x10^6^ cells were collected in 96 well plates via centrifugation, resuspended in 250 μl of 100 ng/ml PI in PBS and incubated for 10 min in the dark. Cells were pelleted, washed once in PBS and subjected to quantification via fluorescence reader (TECAN GeniosPro) or flow cytometry (BD LSR Fortessa/ Guava easyCyte 5HT). For quantifications using flow cytometry, at least 3000 cells were evaluated and analyzed with BD FACSDiva/ InCyte (3.1) software. To determine growth, cells were shifted to SCG at OD_600_ 0.3, transferred to Honeycomb microplates and analyzed using a Bioscreen C (Oy Growth Curves Ab Ltd). Plates were kept shaking at medium speed at 28°C, cell density was determined every 30 min for 24 h and shaking was interrupted 5 sec prior to each measurement. Notably, for all experiments, at least four different clones were tested after gene deletion via homologous recombination and plasmid transformation to rule out clonogenic variations.

### Drosophila stocks, husbandry and climbing activity

All Drosophila lines were kept at 25°C, 60% humidity and a 12 h light/dark cycle on standard potato sucrose food (per liter: 12.9 g dry yeast, 500 ml syrup, 40 g instant mashed potato powder, 10 g agar, 8.5 ml Nipagin and 1 g ascorbic acid). Wild type w^1118^ flies (3605) as well as the UAS-αSyn line (8146) were obtained from Bloomington stock center (Indiana University, USA) and the *nsyb-GAL4* driver line (gift from Stephan Sigrist) was used for pan-neuronal expression. All lines were isogenized against w^1118^ for at least 6 generations. Crossings were performed using a female to male ratio of about 3:1. The genotypes Nsyb-Gal4>w^1118^ and Nsyb-Gal4>UAS-αSyn were used for all experiments, and 1–3 days old flies were collected, sex-sorted using CO_2_ anesthesia and females were transferred onto fresh food in cohorts of 30 flies per vial. Flies were kept at 29°C for 24 h to enhance GAL4-driven expression of the UAS-αSyn constructs prior to transfer to food supplemented or not with additional 15 mM or 30 mM CaCl_2_ for 1 or 2 weeks for Ca^2+^ pre-feeding at 25°C. Subsequently, flies were switched to food containing 10 mM MnCl_2_ (in 10% sucrose and 1% agar) and the number of dead flies was counted every 12 h. Fresh food was provided every second day. All lifespan experiments were repeated at least twice, and the depicted lifespans represent the total number of analyzed flies (exact numbers are indicated in the respective figure legends). In addition, flies were kept on food supplemented with either 15 mM CaCl_2_ or 15 mM MgCl_2_ as a control for two weeks at 25°C for determination of Cathepsin D activity. To assess climbing ability of flies, 6–8 flies were transferred into climbing vials without anesthesia to avoid confounding CO_2_ effects. Flies were allowed to adjust to red light for 20 min before measuring climbing activity. Flies were tapped down and climbing was recorded for 30 s. Three replicate runs were recorded per vial to determine mean climbing activity, and six independent experiments were performed per genotype. For evaluation, freeze images after 3 s of each run were generated, and the covered distance of each fly was analyzed using Fiji.

### Confocal fluorescence microscopy

Subcellular localization of αSyn-GFP in living cells was assessed via confocal microscopy using a ZEISS LSM800 Airyscan microscope (Fig 1C), equipped with a Plan-Apochromat 63x/1.40 Oil M27 objective, and ZEISS ZEN software control. Micrographs in Figs 2B and 3E were recorded with a Leica SP5 confocal laser scanning microscope, equipped with a Leica HCX PL Apo 63x NA 1.4 oil immersion objective. Cells were counterstained with PI to visualize dead cells and subsequently immobilized on agar slides. Micrographs were processed with the open-source software Fiji [105]. Gaussian filtering (σ = 1) was applied to reduce image noise, followed by background subtraction (rolling ball radius = 50 pixels). Pictures within an experiment were captured and processed using the same settings.

### Immunoblotting of yeast and fly lysates

Yeast whole cell extracts were generated using chemical lysis. Briefly, about 3x10^7^ cells were collected, resuspended in 150 μl of 1.85 M NaOH/ 7.5% β-mercaptoethanol and incubated on ice for 10 min. Proteins were precipitated using 150 μl of 55% TCA and incubation on ice for 10 min. Subsequently, protein extracts were centrifuged for 10 min at 10000 g and 4°C, the supernatant was removed and the samples were resuspended in 150 μl urea loading buffer (200 mM Tris/HCl; 8 M urea; 5% SDS; 1 mM EDTA, 0.02% bromophenol blue; 15 mM DTT; pH 6.8) and incubated for 10 min at 65°C prior to loading on standard SDS-PAGE. Immunoblotting was performed using standard protocols with antibodies specific for FLAG-epitope (Sigma; F3165), yeast GAPDH (gift from Günther Daum, TU Graz), and GFP (Roche; #11814460001) and the respective peroxidase-conjugated affinity-purified secondary antibodies (Sigma).

To monitor αSyn dimers, about 8x10^7^ cells were harvested 36 h after induction of galactose-driven expression, resuspended in 200 μl of 0.1 M NaOH and incubated at room temperature for 5 min, shaking with 1400 rpm. After centrifugation with 1500 g for 5 min, samples were resuspended in 150 μl non-reducing Lämmli buffer (50 mM Tris-HCl; 2% SDS; 10% glycerol; 0.1% bromophenol blue; pH 6.8) and again incubated at room temperature for 5 min with 1400 rpm shaking. After a final centrifugation step with 16000 g for 1 min, 10 μl of supernatant were loaded on polyacrylamide gels without SDS. Of note, electrophoresis was performed at 4°C, followed by immunoblotting using standard protocols and decoration with antibodies specific for αSyn (Sigma-Aldrich S3062) and tubulin (Abcam; ab184970). For detection, a ChemiDoc XRS+ (BioRad) was applied, followed by densitometric quantification with ImageLab v 5.2.1 Software (Bio-Rad).

All indicated molecular weights represent the apparent molecular weights (kDa) as determined with a PageRuler prestained protein ladder (Thermo Fisher Scientific) as stated by the manufacture’s migration patterns. For immunoblotting of Drosophila lysates, six fly heads per sample were collected and mechanically lysed in 24 μl fly lysis buffer (50 mM Tris/HCl, 150 mM NaCl, 1% Na-Deoxycholate, 0.1% Triton X-100, cOmplete Protease Inhibitor (Sigma); adjusted to pH 8.0). After centrifugation with 16000 g at 4°C for 10 min, 6 μl of 5 x Lämmli buffer (250 mM Tris/HCL; 20% SDS; 60% glycerol; adjusted to pH 6.8) were admixed to the supernatant and 15 μl of samples were applied for standard SDS-PAGE and immunoblotting. Blots were decorated with antibodies against αSyn (Sigma-Aldrich S3062) and Tubulin (Sigma-Aldrich T9026).

### Total reflection X-ray fluorescence (TXRF) spectrometry

For whole cell multi-element analysis 6x10^7^ cells were harvested by centrifugation (3 min, 3500 g), washed in 300 μl Milli-Q H_2_O, snap frozen in liquid nitrogen and stored at -20°C until further processing. Frozen cell pellets were resuspended in 100 μl 1% Triton X-100 at room temperature, mixed 1:1 with gallium standard solution (2 mg/l) and vortexed. 10 μl of sample were transferred to TXRF quartz glass carriers and carefully dried on a hot plate. Data collection was carried out for 1000 s on an S2 PICOFOX (automatic) spectrometer (Bruker Nano GmbH, Germany) equipped with a molybdenum excitation source (50 kV/600 μA). Elements were assigned manually and spectra quantified in the PICOFOX software. Spectra were recorded for n = 4 biological replicates per condition and values are represented as fold change to WT controls.

### Cathepsin D activity assay

Measurement of Pep4/Cathepsin D activity was performed using a fluorometric Cathepsin D activity assay kit from Abcam (ab65302) according to the manufacturers protocol. For analysis of Pep4 activity in yeast, 2x10^6^ cells were harvested 16 h after induction of galactose-driven expression of αSyn. Protein extracts were generated by mechanical lysis using glass beads and the supplied CD cell lysis buffer. For measurement of Cathepsin D activity in *D*. *melanogaster*, five fly heads per sample were collected at indicated time points and subsequently mechanically lysed in supplied CD cell lysis buffer. Protein concentration was determined via Bradford assay (Bio-Rad) and 0.1 μg protein was used for the Pep4/Cathepsin D activity assay. Reactions for yeast samples were incubated for 2 h at 28°C and fly samples for 2 h at 25°C. Fluorescence signal was measured with a Tecan Genios pro microplate reader (ex. 328 nm, em. 460 nm). Of note, lysates from Δ*pep4* yeast strains or wild type flies treated with 150 μM pepstatin A (dissolved in DMSO) were used as background control.

### Determination of calcineurin activity

Calcineurin activity was determined using cells equipped with the reporter plasmid pAMS366-4xCDRE-GFP^PEST^, which codes for a destabilized GFP protein (yEGFP^PEST^) under the control of a calcineurin-dependent response element (CDRE) as previously described [56]. Briefly, about 2x10^5^ yeast cells were harvested and stained with 100 ng/ml PI in PBS for 7 min. Cells were pelleted, resuspended in PBS and 3000 cells were analyzed using a Guava easyCyte 5HT flow cytometer. PI co-staining served to exclude dead cells from the analysis. Cells lacking Cnb1, the regulatory calcineurin subunit, served as control and obtained fluorescence intensities were subtracted as background.

### Measurement of cytosolic calcium levels

Aequorin-based measurement of cytosolic calcium levels was performed as described earlier [26]. Briefly, the pEVP11/AEQ89 plasmid, coding for the bioluminescent reporter protein aequorin (kind gift from Kyle W. Cunningham) was transformed into yeast cells, which were cultivated as described above. At indicated time points, 1x10^8^ cells were harvested in 96-well plates, resuspended in 200 μl SCD medium containing 4 μM coelenterazine h (ThermoFisher Scientific) and incubated for 1 h in the dark. To remove excess coelenterazine h, cells were washed once in SCD medium and incubated for 30 min. Luminescence signals were recorded in 0.5 s intervals for 25 s (basal Ca^2+^ levels) or 7 s + 80 s (response to external Ca^2+^ pulses) on a GloMax Multi Detection system (Promega). To follow the rapid cellular response to external Ca^2+^ addition, Ca^2+^ was automatically injected after 7 s to final concentrations of 10 mM or 50 mM. Values were normalized to OD_600_.

### Statistical analyses

One-factor analysis of variance (ANOVA) corrected by a Tukey post-hoc test was used for all experiments with the following exceptions: A two-way ANOVA with time and strain as independent factors followed by a Tukey post-hoc test was applied to calculate differences in cytosolic Ca^2+^ levels over time (Fig 2F) and to compare αSyn toxicity in wild type and deletion mutants of the calcineurin pathway over time (Figs 1B and 3B). Statistical analysis for Drosophila survival was performed using Kaplan-Meier survival analysis and pairwise log rank comparisons were corrected via Bonferroni post-hoc test (Fig 6A, 6B, 6E and 6F). For climbing ability, a Kruskal-Wallis test was performed due to non-normally distributed data (Fig 6G).

## Supporting information

S1 Fig Ca^2+^ addition already protects against mild αSyn toxicity during exponential growth. (PDF)

S2 Fig In cells lacking Cnb1, the Ca^2+^-mediated reduction of dimeric αSyn species is mildly impaired. (PDF)

S1 Table Yeast strains used in this study. (PDF)

S2 Table Primers used for gene disruption. (PDF)

## References

[1] Nussbaum RL, Polymeropoulos MH. Genetics of Parkinson’s disease. HumMolGenet. 1997;6: 1687–1691. doi: 10.1093/hmg/6.10.1687 9300660

[2] Pang SY-Y, Ho PW-L, Liu H-F, Leung C-T, Li L, Chang EES, et al. The interplay of aging, genetics and environmental factors in the pathogenesis of Parkinson’s disease. Transl Neurodegener. 2019;8: 23. doi: 10.1186/s40035-019-0165-9 31428316 PMC6696688

[3] Spillantini MG, Schmidt ML, Lee VM, Trojanowski JQ, Jakes R, Goedert M. Alpha-synuclein in Lewy bodies. Nature. 1997;388: 839–840. doi: 10.1038/42166 9278044

[4] Gorell JM, Johnson CC, Rybicki BA, Peterson EL, Kortsha GX, Brown GG, et al. Occupational exposures to metals as risk factors for Parkinson’s disease. Neurology. 1997;48: 650–658. doi: 10.1212/wnl.48.3.650 9065542

[5] Gorell JM, Johnson CC, Rybicki BA, Peterson EL, Kortsha GX, Brown GG, et al. Occupational exposure to manganese, copper, lead, iron, mercury and zinc and the risk of Parkinson’s disease. Neurotoxicology. 1999;20: 239–247. 10385887

[6] Lucchini RG, Guazzetti S, Renzetti S, Broberg K, Caci M, Covolo L, et al. Metal Exposure and SNCA rs356219 Polymorphism Associated With Parkinson Disease and Parkinsonism. Front Neurol. 2020;11: 556337. doi: 10.3389/fneur.2020.556337 33362685 PMC7755861

[7] Rybicki BA, Johnson CC, Uman J, Gorell JM. Parkinson’s disease mortality and the industrial use of heavy metals in Michigan. Mov Disord Off J Mov Disord Soc. 1993;8: 87–92. doi: 10.1002/mds.870080116 8419812

[8] Barnham KJ, Bush AI. Metals in Alzheimer’s and Parkinson’s diseases. Curr Opin Chem Biol. 2008;12: 222–228. doi: 10.1016/j.cbpa.2008.02.019 18342639

[9] Ijomone OM, Ifenatuoha CW, Aluko OM, Ijomone OK, Aschner M. The aging brain: impact of heavy metal neurotoxicity. Crit Rev Toxicol. 2020;50: 801–814. doi: 10.1080/10408444.2020.1838441 33210961

[10] Mezzaroba L, Alfieri DF, Colado Simão AN, Vissoci Reiche EM. The role of zinc, copper, manganese and iron in neurodegenerative diseases. Neurotoxicology. 2019;74: 230–241. doi: 10.1016/j.neuro.2019.07.007 31377220

[11] Farrer M, Kachergus J, Forno L, Lincoln S, Wang DS, Hulihan M, et al. Comparison of kindreds with parkinsonism and alpha-synuclein genomic multiplications. AnnNeurol. 2004;55: 174–179. doi: 10.1002/ana.10846 14755720

[12] Kruger R, Kuhn W, Muller T, Woitalla D, Graeber M, Kosel S, et al. Ala30Pro mutation in the gene encoding alpha-synuclein in Parkinson’s disease. NatGenet. 1998;18: 106–108. doi: 10.1038/ng0298-106 9462735

[13] Polymeropoulos MH, Lavedan C, Leroy E, Ide SE, Dehejia A, Dutra A, et al. Mutation in the alpha-synuclein gene identified in families with Parkinson’s disease. Science. 1997;276: 2045–2047. doi: 10.1126/science.276.5321.2045 9197268

[14] Singleton AB, Farrer M, Johnson J, Singleton A, Hague S, Kachergus J, et al. alpha-Synuclein locus triplication causes Parkinson’s disease. Science. 2003;302: 841. doi: 10.1126/science.1090278 14593171

[15] Zarranz JJ, Alegre J, Gomez-Esteban JC, Lezcano E, Ros R, Ampuero I, et al. The new mutation, E46K, of alpha-synuclein causes Parkinson and Lewy body dementia. AnnNeurol. 2004;55: 164–173. doi: 10.1002/ana.10795 14755719

[16] Harischandra DS, Rokad D, Neal ML, Ghaisas S, Manne S, Sarkar S, et al. Manganese promotes the aggregation and prion-like cell-to-cell exosomal transmission of α-synuclein. Sci Signal. 2019;12. doi: 10.1126/scisignal.aau4543 30862700 PMC6435331

[17] Lorentzon E, Kumar R, Horvath I, Wittung-Stafshede P. Differential effects of Cu2+ and Fe3+ ions on in vitro amyloid formation of biologically-relevant α-synuclein variants. Biometals Int J Role Met Ions Biol Biochem Med. 2020;33: 97–106. doi: 10.1007/s10534-020-00234-4 32170541 PMC7295844

[18] Moons R, Konijnenberg A, Mensch C, Van Elzen R, Johannessen C, Maudsley S, et al. Metal ions shape α-synuclein. Sci Rep. 2020;10: 16293. doi: 10.1038/s41598-020-73207-9 33004902 PMC7529799

[19] Uversky VN, Li J, Fink AL. Metal-triggered structural transformations, aggregation, and fibrillation of human alpha-synuclein. A possible molecular NK between Parkinson’s disease and heavy metal exposure. J Biol Chem. 2001;276: 44284–44296. doi: 10.1074/jbc.M105343200 11553618

[20] Rcom-H’cheo-Gauthier AN, Osborne SL, Meedeniya ACB, Pountney DL. Calcium: Alpha-Synuclein Interactions in Alpha-Synucleinopathies. Front Neurosci. 2016;10: 570. doi: 10.3389/fnins.2016.00570 28066161 PMC5167751

[21] Lautenschläger J, Stephens AD, Fusco G, Ströhl F, Curry N, Zacharopoulou M, et al. C-terminal calcium binding of α-synuclein modulates synaptic vesicle interaction. Nat Commun. 2018;9: 712. doi: 10.1038/s41467-018-03111-4 29459792 PMC5818535

[22] Lowe R, Pountney DL, Jensen PH, Gai WP, Voelcker NH. Calcium(II) selectively induces alpha-synuclein annular oligomers via interaction with the C-terminal domain. Protein Sci Publ Protein Soc. 2004;13: 3245–3252. doi: 10.1110/ps.04879704 15537754 PMC2287302

[23] Nath S, Goodwin J, Engelborghs Y, Pountney DL. Raised calcium promotes α-synuclein aggregate formation. Mol Cell Neurosci. 2011;46: 516–526. doi: 10.1016/j.mcn.2010.12.004 21145971

[24] Rcom-H’cheo-Gauthier AN, Meedeniya ACB, Pountney DL. Calcipotriol inhibits α-synuclein aggregation in SH-SY5Y neuroblastoma cells by a Calbindin-D28k-dependent mechanism. J Neurochem. 2017;141: 263–274. doi: 10.1111/jnc.13971 28164279

[25] Adamczyk A, Strosznajder JB. Alpha-synuclein potentiates Ca2+ influx through voltage-dependent Ca2+ channels. Neuroreport. 2006;17: 1883–1886. doi: 10.1097/WNR.0b013e3280115185 17179863

[26] Büttner S, Faes L, Reichelt WN, Broeskamp F, Habernig L, Benke S, et al. The Ca2+/Mn2+ ion-pump PMR1 links elevation of cytosolic Ca(2+) levels to α-synuclein toxicity in Parkinson’s disease models. Cell Death Differ. 2013;20: 465–477. doi: 10.1038/cdd.2012.142 23154387 PMC3569987

[27] Callewaert G, D’hooge P, Ma T-Y, Del Vecchio M, Van Eyck V, Franssens V, et al. Decreased Vacuolar Ca2+ Storage and Disrupted Vesicle Trafficking Underlie Alpha-Synuclein-Induced Ca2+ Dysregulation in S. cerevisiae. Front Genet. 2020;11: 266. doi: 10.3389/fgene.2020.00266 32457789 PMC7225347

[28] Danzer KM, Haasen D, Karow AR, Moussaud S, Habeck M, Giese A, et al. Different species of alpha-synuclein oligomers induce calcium influx and seeding. J Neurosci Off J Soc Neurosci. 2007;27: 9220–9232. doi: 10.1523/JNEUROSCI.2617-07.2007 17715357 PMC6672196

[29] Hettiarachchi NT, Parker A, Dallas ML, Pennington K, Hung C-C, Pearson HA, et al. alpha-Synuclein modulation of Ca2+ signaling in human neuroblastoma (SH-SY5Y) cells. J Neurochem. 2009;111: 1192–1201. doi: 10.1111/j.1471-4159.2009.06411.x 19860837

[30] Crocker SJ, Smith PD, Jackson-Lewis V, Lamba WR, Hayley SP, Grimm E, et al. Inhibition of calpains prevents neuronal and behavioral deficits in an MPTP mouse model of Parkinson’s disease. J Neurosci Off J Soc Neurosci. 2003;23: 4081–4091. 10.1523/JNEUROSCI.23-10-04081.2003 PMC6741113 12764095

[31] Mishizen-Eberz AJ, Norris EH, Giasson BI, Hodara R, Ischiropoulos H, Lee VM-Y, et al. Cleavage of alpha-synuclein by calpain: potential role in degradation of fibrillized and nitrated species of alpha-synuclein. Biochemistry. 2005;44: 7818–7829. doi: 10.1021/bi047846q 15909996

[32] Angelova PR, Ludtmann MHR, Horrocks MH, Negoda A, Cremades N, Klenerman D, et al. Ca2+ is a key factor in α-synuclein-induced neurotoxicity. J Cell Sci. 2016;129: 1792–1801. doi: 10.1242/jcs.180737 26989132 PMC4893653

[33] Betzer C, Lassen LB, Olsen A, Kofoed RH, Reimer L, Gregersen E, et al. Alpha-synuclein aggregates activate calcium pump SERCA leading to calcium dysregulation. EMBO Rep. 2018;19: e44617. doi: 10.15252/embr.201744617 29599149 PMC5934765

[34] Caraveo G, Auluck PK, Whitesell L, Chung CY, Baru V, Mosharov EV, et al. Calcineurin determines toxic versus beneficial responses to α-synuclein. Proc Natl Acad Sci U S A. 2014;111: E3544–3552. doi: 10.1073/pnas.1413201111 25122673 PMC4151770

[35] Lieberman OJ, Choi SJ, Kanter E, Saverchenko A, Frier MD, Fiore GM, et al. α-Synuclein-Dependent Calcium Entry Underlies Differential Sensitivity of Cultured SN and VTA Dopaminergic Neurons to a Parkinsonian Neurotoxin. eNeuro. 2017;4. doi: 10.1523/ENEURO.0167-17.2017 29177188 PMC5701296

[36] Boland B, Yu WH, Corti O, Mollereau B, Henriques A, Bezard E, et al. Promoting the clearance of neurotoxic proteins in neurodegenerative disorders of ageing. Nat Rev Drug Discov. 2018;17: 660–688. doi: 10.1038/nrd.2018.109 30116051 PMC6456907

[37] Lynch-Day MA, Mao K, Wang K, Zhao M, Klionsky DJ. The role of autophagy in Parkinson’s disease. Cold Spring Harb Perspect Med. 2012;2: a009357. doi: 10.1101/cshperspect.a009357 22474616 PMC3312403

[38] Manzoni C, Lewis PA. Dysfunction of the autophagy/lysosomal degradation pathway is a shared feature of the genetic synucleinopathies. FASEB J. 2013;27: 3424–3429. doi: 10.1096/fj.12-223842 23682122 PMC4194632

[39] Tofaris GK. Lysosome-dependent pathways as a unifying theme in Parkinson’s disease. Mov Disord Off J Mov Disord Soc. 2012;27: 1364–1369. doi: 10.1002/mds.25136 22927213

[40] Tsunemi T, Perez-Rosello T, Ishiguro Y, Yoroisaka A, Jeon S, Hamada K, et al. Increased Lysosomal Exocytosis Induced by Lysosomal Ca2+ Channel Agonists Protects Human Dopaminergic Neurons from α-Synuclein Toxicity. J Neurosci Off J Soc Neurosci. 2019;39: 5760–5772. doi: 10.1523/JNEUROSCI.3085-18.2019 31097622 PMC6636071

[41] Büttner S, Habernig L, Broeskamp F, Ruli D, Vögtle FN, Vlachos M, et al. Endonuclease G mediates α-synuclein cytotoxicity during Parkinson’s disease. EMBO J. 2013;32: 3041–3054. doi: 10.1038/emboj.2013.228 24129513 PMC3844953

[42] Cooper AA, Gitler AD, Cashikar A, Haynes CM, Hill KJ, Bhullar B, et al. Alpha-synuclein blocks ER-Golgi traffic and Rab1 rescues neuron loss in Parkinson’s models. Science. 2006;313: 324–328. doi: 10.1126/science.1129462 16794039 PMC1983366

[43] Feany MB, Bender WW. A Drosophila model of Parkinson’s disease. Nature. 2000;404: 394–398. doi: 10.1038/35006074 10746727

[44] Gitler AD, Chesi A, Geddie ML, Strathearn KE, Hamamichi S, Hill KJ, et al. Alpha-synuclein is part of a diverse and highly conserved interaction network that includes PARK9 and manganese toxicity. Nat Genet. 2009;41: 308–315. doi: 10.1038/ng.300 19182805 PMC2683786

[45] Outeiro TF, Lindquist S. Yeast cells provide insight into alpha-synuclein biology and pathobiology. Science. 2003;302: 1772–1775. doi: 10.1126/science.1090439 14657500 PMC1780172

[46] Bagur R, Hajnóczky G. Intracellular Ca2+ Sensing: Its Role in Calcium Homeostasis and Signaling. Mol Cell. 2017;66: 780–788. doi: 10.1016/j.molcel.2017.05.028 28622523 PMC5657234

[47] Cui J, Kaandorp JA, Sloot PMA, Lloyd CM, Filatov MV. Calcium homeostasis and signaling in yeast cells and cardiac myocytes. FEMS Yeast Res. 2009;9: 1137–1147. doi: 10.1111/j.1567-1364.2009.00552.x 19678847

[48] Cyert MS, Philpott CC. Regulation of cation balance in Saccharomyces cerevisiae. Genetics. 2013;193: 677–713. doi: 10.1534/genetics.112.147207 23463800 PMC3583992

[49] Binolfi A, Rasia RM, Bertoncini CW, Ceolin M, Zweckstetter M, Griesinger C, et al. Interaction of alpha-synuclein with divalent metal ions reveals key differences: a link between structure, binding specificity and fibrillation enhancement. J Am Chem Soc. 2006;128: 9893–9901. doi: 10.1021/ja0618649 16866548

[50] Choi TS, Lee J, Han JY, Jung BC, Wongkongkathep P, Loo JA, et al. Supramolecular Modulation of Structural Polymorphism in Pathogenic α-Synuclein Fibrils Using Copper(II) Coordination. Angew Chem Int Ed. 2018;57: 3099–3103. doi: 10.1002/anie.201712286 29368447

[51] Santner A, Uversky VN. Metalloproteomics and metal toxicology of α-synuclein. Met Integr Biometal Sci. 2010;2: 378–392. doi: 10.1039/b926659c 21072383

[52] Wright JA, Brown DR. Alpha-synuclein and its role in metal binding: relevance to Parkinson’s disease. J Neurosci Res. 2008;86: 496–503. doi: 10.1002/jnr.21461 17705291

[53] Follett J, Darlow B, Wong MB, Goodwin J, Pountney DL. Potassium depolarization and raised calcium induces α-synuclein aggregates. Neurotox Res. 2013;23: 378–392. doi: 10.1007/s12640-012-9366-z 23250862

[54] Han JY, Choi TS, Kim HI. Molecular Role of Ca2+ and Hard Divalent Metal Cations on Accelerated Fibrillation and Interfibrillar Aggregation of α-Synuclein. Sci Rep. 2018;8: 1895. doi: 10.1038/s41598-018-20320-5 29382893 PMC5789889

[55] Connolly S, Kingsbury T. Regulatory Subunit Myristoylation Antagonizes Calcineurin Phosphatase Activation in Yeast. J Biol Chem. 2012;287: 39361–39368. doi: 10.1074/jbc.M112.366617 23027860 PMC3501033

[56] Diessl J, Nandy A, Schug C, Habernig L, Büttner S. Stable and destabilized GFP reporters to monitor calcineurin activity in Saccharomyces cerevisiae. Microb Cell Graz Austria. 2020;7: 106–114. doi: 10.15698/mic2020.04.713 32274389 PMC7136757

[57] Berridge MJ, Lipp P, Bootman MD. The versatility and universality of calcium signalling. Nat Rev Mol Cell Biol. 2000;1: 11–21. doi: 10.1038/35036035 11413485

[58] Matheos DP, Kingsbury TJ, Ahsan US, Cunningham KW. Tcn1p/Crz1p, a calcineurin-dependent transcription factor that differentially regulates gene expression in Saccharomyces cerevisiae. Genes Dev. 1997;11: 3445–3458. doi: 10.1101/gad.11.24.3445 9407036 PMC316804

[59] Stathopoulos-Gerontides A, Guo JJ, Cyert MS. Yeast calcineurin regulates nuclear localization of the Crz1p transcription factor through dephosphorylation. Genes Dev. 1999;13: 798–803. doi: 10.1101/gad.13.7.798 10197980 PMC316598

[60] Goldman A, Roy J, Bodenmiller B, Wanka S, Landry CR, Aebersold R, et al. The calcineurin signaling network evolves via conserved kinase-phosphatase modules that transcend substrate identity. Mol Cell. 2014;55: 422–435. doi: 10.1016/j.molcel.2014.05.012 24930733 PMC4127121

[61] Piña FJ, O’Donnell AF, Pagant S, Piao HL, Miller JP, Fields S, et al. Hph1 and Hph2 are novel components of the Sec63/Sec62 posttranslational translocation complex that aid in vacuolar proton ATPase biogenesis. Eukaryot Cell. 2011;10: 63–71. doi: 10.1128/EC.00241-10 21097665 PMC3019806

[62] Spedale G, Mischerikow N, Heck AJR, Timmers HTM, Pijnappel WWMP. Identification of Pep4p as the protease responsible for formation of the SAGA-related SLIK protein complex. J Biol Chem. 2010;285: 22793–22799. doi: 10.1074/jbc.M110.108787 20498363 PMC2906270

[63] Kerstens W, Dijck PV. A Cinderella story: how the vacuolar proteases Pep4 and Prb1 do more than cleaning up the cell’s mass degradation processes. Microb Cell. 2018;5: 438–443. doi: 10.15698/mic2018.10.650 30386788 PMC6206407

[64] D’hooge P, Coun C, Van Eyck V, Faes L, Ghillebert R, Mariën L, et al. Ca(2+) homeostasis in the budding yeast Saccharomyces cerevisiae: Impact of ER/Golgi Ca(2+) storage. Cell Calcium. 2015;58: 226–235. doi: 10.1016/j.ceca.2015.05.004 26055636

[65] Strayle J, Pozzan T, Rudolph HK. Steady-state free Ca(2+) in the yeast endoplasmic reticulum reaches only 10 microM and is mainly controlled by the secretory pathway pump pmr1. EMBO J. 1999;18: 4733–4743. doi: 10.1093/emboj/18.17.4733 10469652 PMC1171546

[66] Aufschnaiter A, Habernig L, Kohler V, Diessl J, Carmona-Gutierrez D, Eisenberg T, et al. The Coordinated Action of Calcineurin and Cathepsin D Protects Against α-Synuclein Toxicity. Front Mol Neurosci. 2017;10: 207. doi: 10.3389/fnmol.2017.00207 28713240 PMC5491553

[67] Uribe S, Rangel P, Pardo JP. Interactions of calcium with yeast mitochondria. Cell Calcium. 1992;13: 211–217. doi: 10.1016/0143-4160(92)90009-h 1586938

[68] Kovács-Bogdán E, Sancak Y, Kamer KJ, Plovanich M, Jambhekar A, Huber RJ, et al. Reconstitution of the mitochondrial calcium uniporter in yeast. Proc Natl Acad Sci U S A. 2014;111: 8985–8990. doi: 10.1073/pnas.1400514111 24889638 PMC4066498

[69] Zulkifli M, Neff JK, Timbalia SA, Garza NM, Chen Y, Watrous JD, et al. Yeast homologs of human MCUR1 regulate mitochondrial proline metabolism. Nat Commun. 2020;11: 4866. doi: 10.1038/s41467-020-18704-1 32978391 PMC7519068

[70] Halachmi D, Eilam Y. Elevated cytosolic free Ca2+ concentrations and massive Ca2+ accumulation within vacuoles, in yeast mutant lacking PMR1, a homolog of Ca2+ -ATPase. FEBS Lett. 1996;392: 194–200. doi: 10.1016/0014-5793(96)00799-5 8772202

[71] Cunningham KW, Fink GR. Calcineurin inhibits VCX1-dependent H+/Ca2+ exchange and induces Ca2+ ATPases in Saccharomyces cerevisiae. Mol Cell Biol. 1996;16: 2226–2237. doi: 10.1128/MCB.16.5.2226 8628289 PMC231210

[72] Matrone C, Dzamko N, Madsen P, Nyegaard M, Pohlmann R, Søndergaard RV, et al. Mannose 6-Phosphate Receptor Is Reduced in -Synuclein Overexpressing Models of Parkinsons Disease. PloS One. 2016;11: e0160501. doi: 10.1371/journal.pone.0160501 27509067 PMC4979956

[73] Sevlever D, Jiang P, Yen S-HC. Cathepsin D is the main lysosomal enzyme involved in the degradation of alpha-synuclein and generation of its carboxy-terminally truncated species. Biochemistry. 2008;47: 9678–9687. doi: 10.1021/bi800699v 18702517 PMC2630205

[74] Qiao L, Hamamichi S, Caldwell KA, Caldwell GA, Yacoubian TA, Wilson S, et al. Lysosomal enzyme cathepsin D protects against alpha-synuclein aggregation and toxicity. MolBrain. 2008;1: 17. doi: 10.1186/1756-6606-1-17 19021916 PMC2600785

[75] Zimprich A, Benet-Pagès A, Struhal W, Graf E, Eck SH, Offman MN, et al. A mutation in VPS35, encoding a subunit of the retromer complex, causes late-onset Parkinson disease. Am J Hum Genet. 2011;89: 168–175. doi: 10.1016/j.ajhg.2011.06.008 21763483 PMC3135812

[76] McMillan KJ, Gallon M, Jellett AP, Clairfeuille T, Tilley FC, McGough I, et al. Atypical parkinsonism-associated retromer mutant alters endosomal sorting of specific cargo proteins. J Cell Biol. 2016;214: 389–399. doi: 10.1083/jcb.201604057 27528657 PMC4987296

[77] Williams ET, Chen X, Moore DJ. VPS35, the Retromer Complex and Parkinson’s Disease. J Park Dis. 2017;7: 219–233. doi: 10.3233/JPD-161020 28222538 PMC5438477

[78] Aufschnaiter A, Kohler V, Büttner S. Taking out the garbage: cathepsin D and calcineurin in neurodegeneration. Neural Regen Res. 2017;12: 1776–1779. doi: 10.4103/1673-5374.219031 29239314 PMC5745822

[79] Follett J, Norwood SJ, Hamilton NA, Mohan M, Kovtun O, Tay S, et al. The Vps35 D620N mutation linked to Parkinson’s disease disrupts the cargo sorting function of retromer. Traffic Cph Den. 2014;15: 230–244. doi: 10.1111/tra.12136 24152121

[80] Kweon Y, Rothe A, Conibear E, Stevens TH. Ykt6p is a multifunctional yeast R-SNARE that is required for multiple membrane transport pathways to the vacuole. Mol Biol Cell. 2003;14: 1868–1881. doi: 10.1091/mbc.e02-10-0687 12802061 PMC165083

[81] McGrath K, Agarwal S, Tonelli M, Dergai M, Gaeta AL, Shum AK, et al. A conformational switch driven by phosphorylation regulates the activity of the evolutionarily conserved SNARE Ykt6. Proc Natl Acad Sci U S A. 2021;118: e2016730118. doi: 10.1073/pnas.2016730118 33723042 PMC8000380

[82] Sakata N, Shirakawa R, Goto K, Trinh DA, Horiuchi H. Double prenylation of SNARE protein Ykt6 is required for lysosomal hydrolase trafficking. J Biochem (Tokyo). 2021;169: 363–370. doi: 10.1093/jb/mvaa111 33035318

[83] Foster TC, Sharrow KM, Masse JR, Norris CM, Kumar A. Calcineurin Links Ca2+ Dysregulation with Brain Aging. J Neurosci. 2001;21: 4066–4073. doi: 10.1523/JNEUROSCI.21-11-04066.2001 11356894 PMC1201477

[84] Graef IA, Wang F, Charron F, Chen L, Neilson J, Tessier-Lavigne M, et al. Neurotrophins and netrins require calcineurin/NFAT signaling to stimulate outgrowth of embryonic axons. Cell. 2003;113: 657–670. doi: 10.1016/s0092-8674(03)00390-8 12787506

[85] Sklar EM. Post-transplant Neurotoxicity: What Role do Calcineurin Inhibitors Actually Play? Am J Neuroradiol. 2006;27: 1602–1603. 16971594 PMC8139761

[86] Wu H-Y, Tomizawa K, Oda Y, Wei F-Y, Lu Y-F, Matsushita M, et al. Critical Role of Calpain-mediated Cleavage of Calcineurin in Excitotoxic Neurodegeneration. J Biol Chem. 2004;279: 4929–4940. doi: 10.1074/jbc.M309767200 14627704

[87] Zeng H, Chattarji S, Barbarosie M, Rondi-Reig L, Philpot BD, Miyakawa T, et al. Forebrain-Specific Calcineurin Knockout Selectively Impairs Bidirectional Synaptic Plasticity and Working/Episodic-like Memory. Cell. 2001;107: 617–629. doi: 10.1016/s0092-8674(01)00585-2 11733061

[88] Shi X, Sun Y, Wang P, Gu L, Wang L, Yang H, et al. The interaction between calcineurin and α-synuclein is regulated by calcium and calmodulin. Biochem Biophys Res Commun. 2018;496: 1109–1114. doi: 10.1016/j.bbrc.2018.01.148 29409956

[89] Calì T, Ottolini D, Brini M. Calcium signaling in Parkinson’s disease. Cell Tissue Res. 2014;357: 439–454. doi: 10.1007/s00441-014-1866-0 24781149

[90] Chan CS, Guzman JN, Ilijic E, Mercer JN, Rick C, Tkatch T, et al. “Rejuvenation” protects neurons in mouse models of Parkinson’s disease. Nature. 2007;447: 1081–1086. doi: 10.1038/nature05865 17558391

[91] Surmeier DJ, Guzman JN, Sanchez-Padilla J. Calcium, cellular aging, and selective neuronal vulnerability in Parkinson’s disease. Cell Calcium. 2010;47: 175–182. doi: 10.1016/j.ceca.2009.12.003 20053445 PMC3235732

[92] Striessnig J, Koschak A, Sinnegger-Brauns MJ, Hetzenauer A, Nguyen NK, Busquet P, et al. Role of voltage-gated L-type Ca2+ channel isoforms for brain function. Biochem Soc Trans. 2006;34: 903–909. doi: 10.1042/BST0340903 17052224

[93] Wilson CJ, Callaway JC. Coupled oscillator model of the dopaminergic neuron of the substantia nigra. J Neurophysiol. 2000;83: 3084–3100. doi: 10.1152/jn.2000.83.5.3084 10805703

[94] Guzman JN, Sanchez-Padilla J, Wokosin D, Kondapalli J, Ilijic E, Schumacker PT, et al. Oxidant stress evoked by pacemaking in dopaminergic neurons is attenuated by DJ-1. Nature. 2010;468: 696–700. doi: 10.1038/nature09536 21068725 PMC4465557

[95] Ilijic E, Guzman J, Surmeier D. The L-type channel antagonist isradipine is neuroprotective in a mouse model of Parkinson’s disease. Neurobiol Dis. 2011;43: 364–371. doi: 10.1016/j.nbd.2011.04.007 21515375 PMC3235730

[96] Parkinson Study Group STEADY-PD III Investigators. Isradipine Versus Placebo in Early Parkinson Disease: A Randomized Trial. Ann Intern Med. 2020;172: 591–598. doi: 10.7326/M19-2534 32227247 PMC7465126

[97] Abou-Raya S, Helmii M, Abou-Raya A. Bone and mineral metabolism in older adults with Parkinson’s disease. Age Ageing. 2009;38: 675–680. doi: 10.1093/ageing/afp137 19684354

[98] Harischandra DS, Jin H, Anantharam V, Kanthasamy A, Kanthasamy AG. α-Synuclein protects against manganese neurotoxic insult during the early stages of exposure in a dopaminergic cell model of Parkinson’s disease. Toxicol Sci Off J Soc Toxicol. 2015;143: 454–468. doi: 10.1093/toxsci/kfu247 25416158 PMC4306724

[99] Yan D-Y, Liu C, Tan X, Ma Z, Wang C, Deng Y, et al. Mn-Induced Neurocytes Injury and Autophagy Dysfunction in Alpha-Synuclein Wild-Type and Knock-Out Mice: Highlighting the Role of Alpha-Synuclein. Neurotox Res. 2019;36: 66–80. doi: 10.1007/s12640-019-00016-y 30796692

[100] Bornhorst J, Chakraborty S, Meyer S, Lohren H, Brinkhaus SG, Knight AL, et al. The effects of pdr1, djr1.1 and pink1 loss in manganese-induced toxicity and the role of α-synuclein in C. elegans. Met Integr Biometal Sci. 2014;6: 476–490. doi: 10.1039/c3mt00325f 24452053 PMC3954834

[101] Gietz RD, Woods RA. Transformation of yeast by lithium acetate/single-stranded carrier DNA/polyethylene glycol method. Methods Enzymol. 2002;350: 87–96. doi: 10.1016/s0076-6879(02)50957-5 12073338

[102] Büttner S, Bitto A, Ring J, Augsten M, Zabrocki P, Eisenberg T, et al. Functional mitochondria are required for alpha-synuclein toxicity in aging yeast. JBiolChem. 2008;283: 7554–7560. doi: 10.1074/jbc.M708477200 18192273

[103] Janke C, Magiera MM, Rathfelder N, Taxis C, Reber S, Maekawa H, et al. A versatile toolbox for PCR-based tagging of yeast genes: new fluorescent proteins, more markers and promoter substitution cassettes. Yeast Chichester Engl. 2004;21: 947–962. doi: 10.1002/yea.1142 15334558

[104] Herker E, Jungwirth H, Lehmann KA, Maldener C, Frohlich KU, Wissing S, et al. Chronological aging leads to apoptosis in yeast. JCell Biol. 2004;164: 501–507. doi: 10.1083/jcb.200310014 14970189 PMC2171996

[105] Schindelin J, Arganda-Carreras I, Frise E, Kaynig V, Longair M, Pietzsch T, et al. Fiji: an open-source platform for biological-image analysis. Nat Methods. 2012;9: 676–682. doi: 10.1038/nmeth.2019 22743772 PMC3855844

