# Supplementary material for: Ca2+ administration prevents α-synuclein proteotoxicity by stimulating calcineurin-dependent lysosomal proteolysis
Source: PLoS Genet. 2021 Nov 15;17(11):e1009911. doi: 10.1371/journal.pgen.1009911 (PMC8629384; doi:10.1371/journal.pgen.1009911)
Supplement: S1 Fig — (PDF) [file pgen.1009911.s001.pdf]

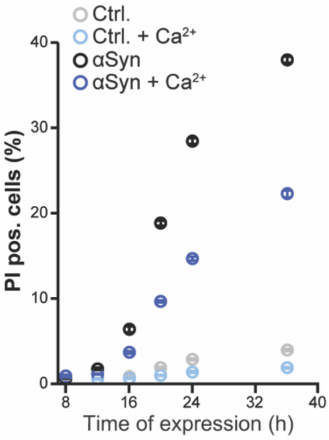

S1 Fig. Ca^{2+} addition already protects against mild αSyn toxicity during exponential growth. Flow cytometric quantification of loss of membrane integrity via propidium iodide (PI) staining in cells expressing αSyn or harboring the vector control at indicated time points. Cells were supplemented with additional 10 mM $CaCl_2$ at the time point of shift to galactose or left untreated. Means ± s.e.m; n=6.
