# Supplementary material for: Ca2+ administration prevents α-synuclein proteotoxicity by stimulating calcineurin-dependent lysosomal proteolysis
Source: PLoS Genet. 2021 Nov 15;17(11):e1009911. doi: 10.1371/journal.pgen.1009911 (PMC8629384; doi:10.1371/journal.pgen.1009911)
Supplement: S2 Fig — (PDF) [file pgen.1009911.s002.pdf]

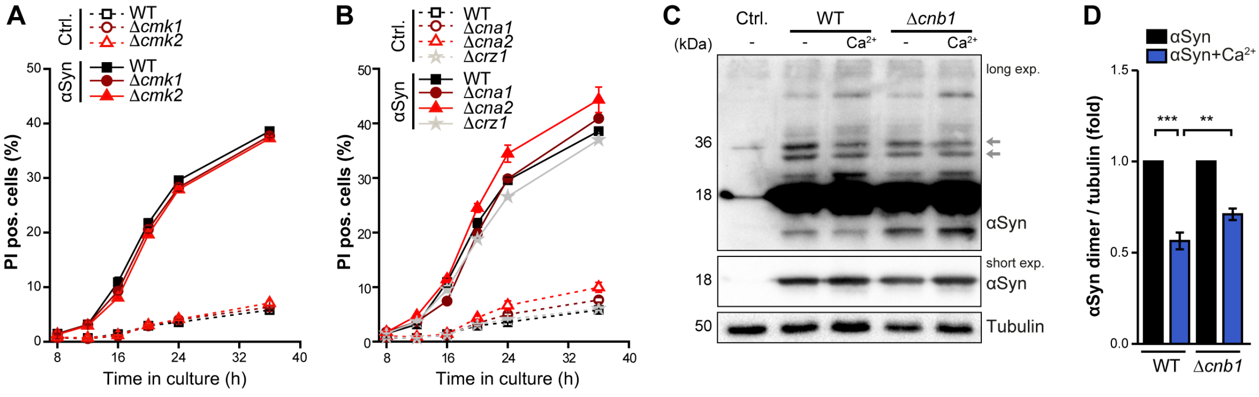

S2 Fig. In cells lacking Cnb1, the Ca^{2+}-mediated reduction of dimeric αSyn species is mildly impaired.

(**A, B**) Flow cytometric quantification of cell death determined by propidium iodide (PI) staining of WT cells and indicated deletion mutants of the Ca^{2+}/calmodulin pathway expressing αSyn or harboring the vector control (Ctrl.) at indicated time points. Means ± s.e.m; n=4.

(**C, D**) Representative semi-native immunoblots of protein extracts of WT and Δ*cnb1* cells expressing αSyn grown on media with and without additional 10 mM Ca^{2+} for 36 h (C) and corresponding densitometric quantification (D) of dimeric αSyn species, as indicated by arrows in (C). Blots were probed with antibodies directed against αSyn (short and long exposure is shown) and tubulin as loading control, and the combined signal of the dimeric αSyn species was normalized to tubulin. Means ± s.e.m; n=5.
