# Supplementary material for: Ca2+ administration prevents α-synuclein proteotoxicity by stimulating calcineurin-dependent lysosomal proteolysis
Source: PLoS Genet. 2021 Nov 15;17(11):e1009911. doi: 10.1371/journal.pgen.1009911 (PMC8629384; doi:10.1371/journal.pgen.1009911)
Supplement: S1 Table — (PDF) [file pgen.1009911.s003.pdf]

S1 Table. Yeast strains used in this study.

Strain	Genotype	Source
BY4741 (WT)	Mata; his3Δ1; leu2Δ0; met15Δ0; ura3Δ0	Euroscarf
Δ*aim3*	BY4741 aim3Δ::kanMX4	Euroscarf
Δ*aly1*	BY4741 aly1Δ::hphNT1	This study
Δ*aly2*	BY4741 aly2Δ::hphNT1	This study
Δ*aly1*Δ*aly2*	BY4741 aly1Δ::kanMX4; aly2Δ::hphNT1	[1]
Δ*caf120*	BY4741 caf120Δ::hphNT1	This study
Δ*skg3*	BY4741 skg3Δ::hphNT1	This study
Δ*skg3*Δ*caf120*	BY4741 skg3Δ::kanMX4; caf120Δ::hphNT1	[1]
Δ*cmk1*	BY4741 cmk1Δ::hphNT1	This study
Δ*cmk2*	BY4741 cmk2Δ::hphNT1	This study
Δ*cna1*	BY4741 cna1Δ::hphNT1	This study
Δ*cna2*	BY4741 cna2Δ::hphNT1	This study
Δ*cna1*Δ*cna2*	BY4741 cna1Δ::kanMX4; cna2Δ::hphNT1	[1]
Δ*cnb1*	BY4741 cnb1Δ::hphNT1	[1]
Δ*crz1*	BY4741 crz1Δ::hphNT1	[1]
Δ*tda1*	BY4741 tda1Δ:: kanMX4	Euroscarf
Δ*hph1*Δ*hph2*	BY4741 hph1Δ::kanMX4; hph2Δ::hphNT1	[1]
Δ*inp52*	BY4741 inp52Δ::hphNT1	This study
Δ*inp53*	BY4741 inp53Δ::hphNT1	This study
Δ*inp52*Δ*inp53*	BY4741 inp52Δ::kanMX4; inp53Δ::hphNT1	[1]
Δ*jip4*	BY4741 jip4Δ::hphNT1	This study
Δ*yor019w*	BY4741 yor019wΔ::hphNT1	This study
Δ*jip4*Δ*yor019w*	BY4741 jip4Δ::hphNT1; yor019wΔ::kanMX4	[1]
Δ*nup60*	BY4741 nup60Δ::kanMX4	Euroscarf
Δ*pep4*	BY4741 pep4Δ::kanMX4	Euroscarf
Δ*pmc1*	BY4741 pmc1Δ::kanMX4	Euroscarf
Δ*vcx1*	BY4741 vcx1Δ::hphNT1	This study

Δ*yvc1*	BY4741 yvc1Δ::hphNT1	[1]
Δ*pmc1*Δ*vcx1*	BY4741 pmc1Δ::kanMX4; vcx1Δ::hphNT1	This study
Δ*pmc1*Δ*yvc1*	BY4741 pmc1Δ::kanMX4; yvc1Δ::hphNT1	This study
Δ*vcx1*Δ*yvc1*	BY4741 vcx1Δ::hphNT1; yvc1Δ::kanMX4	This study
Δ*rcn1*	BY4741 rcn1Δ::hphNT1	This study
Δ*rcn2*	BY4741 rcn2Δ::hphNT1	This study
Δ*rcn1*Δ*rcn2*	BY4741 rcn1Δ::kanMX; rcn2Δ::hphNT1	This study
Δ*rod1*	BY4741 rod1Δ::hphNT1	This study
Δ*rog3*	BY4741 rog3Δ::hphNT1	This study
Δ*rod1*Δ*rog3*	BY4741 rod1Δ::kanMX; rog3Δ::hphNT1	[1]
Δ*sl*a1	BY4741 sla1Δ::kanMX4	Euroscarf
Δ*slm1*	BY4741 slm1Δ::hphNT1	This study
Δ*slm2*	BY4741 slm2Δ::hphNT1	This study
Δ*spt8*	BY4741 spt8Δ::hphNT1	This study
Δ*tfb6*	BY4741 tfb6Δ::kanMX4	Euroscarf
Δ*ubx6*	BY4741 ubx6Δ::hphNT1	This study
Δ*ubx7*	BY4741 ubx7Δ::hphNT1	This study
Δ*yap1*	BY4741 yap1Δ::hphNT1	This study
Δ*cad1*	BY4741 cad1Δ::hphNT1	This study
Δ*yap1*Δ*cad1*	BY4741 yap1Δ::kanMX4; cad1Δ::hphNT1	[1]
Δ*ybr225w*	BY4741 ybr225wΔ::kanMX4	Euroscarf
Δ*ypt6*	BY4741 ypt6Δ::hphNT1	This study
Δ*zrg17*	BY4741 zrg17Δ::kanMX4	Euroscarf
Δ*pmr1*	BY4741 pmr1Δ::kanMX4 HUR1	This study

References

1. Aufschnaiter A, Habernig L, Kohler V, Diessl J, Carmona-Gutierrez D, Eisenberg T, et al. The Coordinated Action of Calcineurin and Cathepsin D Protects Against α-Synuclein Toxicity. Front Mol Neurosci. 2017;10: 207. doi:10.3389/fnmol.2017.00207
