# Supplementary material for: Ca2+ administration prevents α-synuclein proteotoxicity by stimulating calcineurin-dependent lysosomal proteolysis
Source: PLoS Genet. 2021 Nov 15;17(11):e1009911. doi: 10.1371/journal.pgen.1009911 (PMC8629384; doi:10.1371/journal.pgen.1009911)
Supplement: S2 Table — (PDF) [file pgen.1009911.s004.pdf]

**S2 Table. Primers used for gene disruption.**

| Target | Primers | PCR template |
|---|---|---|
| *ALY1* deletion | 5'- CACAAATACGCTGGAATCCCATAGAATATTGCTTGTTCCTCTATG ACTACATGCGTACGCTGCAGGTCGAC -3'<br>5'- GGACACAAAGTAAAATGGTAGCAATTCTGTCGGAGAATGAAAATA ATGTACTTAATCGATGAATTCGAGCTCG -3' | pFA6a–hphNT1 |
| Control PCR *ALY1* | 5'- CACCGAACACTTCCTTCGAG -3'<br>5'- GTCGACCTGCAGCGTACG -3' | |
| *ALY2* deletion | 5'- CGTTGAAACATCTTTTTTTTTTTTCCAGCCTTTCTCATTGTAGATC AGTCATTTTTCAAGATGCGTACGCTGCAGGTCGAC -3'<br>5'- GACGCCAGATGTAAGAACAGGAAGGGATGACGTAGGAACCTTGTGC TAGACTAATCGATGAATTCGAGCTCG -3' | pFA6a–hphNT1 |
| Control PCR *ALY2* | 5'- CATCTCATCCCACTGTTTCATTC -3'<br>5'- GTCGACCTGCAGCGTACG -3' | |
| *CAF120* deletion | 5'- CGGTTTCCCCACACAAAGAACACGTTACTTGGCAAATTCAGCTCTT ATGCGTACGCTGCAGGTCGAC -3'<br>5'- CTTTTCTTAATTGTCTCTCTTTTTGTAAAATTACAACCAGTCGGTC GAGTTAATCGATGAATTCGAGCTCG -3' | pFA6a–hphNT1 |
| Control PCR *CAF120* | 5'- GCCACAGAAAACTTACTGGAG -3'<br>5'- GTCGACCTGCAGCGTACG -3' | |
| *SKG3* deletion | 5'- GGCAATAAAAGAACAAGTAACTGCAAGAACACCAAGAAGCTACAGT AAGAAATGCGTACGCTGCAGGTCGAC -3'<br>5'- CTACTGAATTGTAAAAAAAAAAAAAAAGAAAAGATGAAATATAAATG AATCAATCGATGAATTCGAGCTCG -3' | pFA6a–hphNT1 |
| Control PCR *SKG3* | 5'- GCCATGGCGCTGAAGATGCTTG -3'<br>5'- GTCGACCTGCAGCGTACG -3' | |
| *CMK1* deletion | 5'- TATATAATATTGGAAGACACCAGAAAAAATAACGAGTCAATTACT ATGCGTACGCTGCAGGTCGAC -3'<br>5'- GTCATTGAAGATTTATTCGCCAGTGTGGTAAAACGGCATACTGTTATCA ATCGATGAATTCGAGCTCG -3' | pFA6a–hphNT1 |
| Control PCR *CMK1* | 5'- GTACCCTTCAAGATGCTAAT -3'<br>5'- GTCGACCTGCAGCGTACG -3' | |
| *CMK2* deletion | 5'- CACCTTTTCTTCTATCACATCGCCAATATAAATATA GACACCAAAAATGCGTACGCTGCAGGTCGAC -3'<br>5'-TTAAATATTATATACGAATTTATGTACACGAATTCAAGTCCGTAATTT AATCGATGAATTCGAGCTCG -3' | pFA6a–hphNT1 |
| Control PCR *CMK2* | 5'- TGATGCTTTC GAAGCCTGGT -3'<br>5'- GTCGACCTGCAGCGTACG -3' | |
| *CNA1* deletion | 5'- TTTTGACGTATTAGCTCAGCTGCCATAAAACACTCTCAACGCCAATGCGTA CGCTGCAGGTCGAC -3'<br>5'- ATTCGATGTTGCATCTCATAGGAATAAAAAAGAATAAAATGAGATTTATC AATCGATGAATTCGAGCTCG -3' | pFA6a–hphNT1 |
| Control PCR *CNA1* | 5'- GAACGTACTACTGGGAAACAAAAG -3'<br>5'- GTCGACCTGCAGCGTACG-3' | |

| | | |
|---|---|---|
| *CNA2* deletion | 5'- TTCCCTCCCATAGAGTCACACAGGAGCCAGTACTTCTTCTTGAACCCGCA ATGCGTACGCTGCAGGTCGAC -3'<br>5'- CTTACTTACTTATTGAAGTATGTACAGTGGAAATAGGAGCTTTCTCTAAT CGATGAATTCGAGCTCG -3' | pFA6a–hphNT1 |
| Control PCR *CNA2* | 5'- CCGAGACAAATGAGAAAATGTCC -3'<br>5'- GTCGACCTGCAGCGTACG -3' | |
| *CNB1* deletion | 5'- TGGTAACTCAATGGTGATCAGAATCCATAGAAGCATTTTTATTTCTTAAA ATGCGTACGCTGCAGGTCGAC -3'<br>5'- CTTAAAAATATTGGCATACCATAAATGAATGAAGTGTCCCCTAGTCTTAA TCGATGAATTCGAGCTCG -3' | pFA6a–hphNT1 |
| Control PCR *CNB1* | 5'- GCCGCCAAAATGGGATATAC -3'<br>5'- GTCGACCTGCAGCGTACG -3' | |
| *CRZ1* deletion | 5'- GTATTTTAGTCTCGATTGGAAGTTTCGTCAGACAGTACA AGGAAGATGCGTACGCTGCAGGTCGAC -3'<br>5'- TTATATAGAAAAAAAAAAATTCCTATTCAAAGCTTAAAA AAACAAAAATAATTAATCGATGAATTCGAGCTCG -3' | pFA6a–hphNT1 |
| Control PCR *CRZ1* | 5'- GCGAGCTATATATCAGCGATAC -3'<br>5'- GTCGACCTGCAGCGTACG -3' | |
| *HPH2* deletion | 5'- GAATAACACAAATTGATGGCAGTTTTTTACGTAGTCCAGTAGTTGT CCAGGTACAATGCGTACGCTGCAGGTCGAC -3'<br>5'- CATATGAAAAAATCACAGGATCATTTTTTGATATACAAATACTATT TTTTAATCGATGAATTCGAGCTCG -3' | pFA6a–hphNT1 |
| Control PCR *HPH2* | 5'- GAGACCCTATTCTCATCTAC -3'<br>5'- GTCGACCTGCAGCGTACG -3' | |
| *INP52* deletion | 5'- GGCCACGCAAAGGCAGCAGAATCAAAAACAAATACTCAGTAGCTAT GCGTACGCTGCAGGTCGAC -3'<br>5'- CTAAACAACAGTTATGATACATATTCTATAAATGCGTAATTTTAGT AACACAATTTAATCGATGAATTCGAGCTCG -3' | pFA6a–hphNT1 |
| Control PCR *INP52* | 5'- GCGGCATCTGGCTCTGCATA -3'<br>5'- GTCGACCTGCAGCGTACG -3' | |
| *INP53* deletion | 5'- GAAAATAACTGGGGCGAAGAATATCTAGTTATCCACTCCTTCATAG AATGCGTACGCTGCAGGTCGAC -3'<br>5'- GGGATACAAACGGAACAACAACCACACTTCAAAGATAACATATTCT CAATCGATGAATTCGAGCTCG -3' | pFA6a–hphNT1 |
| Control PCR *INP53* | 5'- GGGGATGTTCTACTGGCAGA -3'<br>5'- GTCGACCTGCAGCGTACG -3' | |
| *JIP4* deletion | 5'- GGAATCTTTAAAGTACAAGGAACAGAAGATACTAAAACATAGGGGG AAATGCGTACGCTGCAGGTCGAC -3'<br>5'- GTTATAGAATTATATCGAATAAACACATAAGAACGTAAGACCAACTAAT CGATGAATTCGAGCTCG -3' | pFA6a–hphNT1 |
| Control PCR *JIP4* | 5'- GAGTGGCTTCTTCTAGGTCTC -3'<br>5'- GTCGACCTGCAGCGTACG -3' | |
| *YOR019W* deletion | 5'- CAACTTGCTATAGATCACGCACCCTTTTGGCAAGTCAACCTTAAAT TATTATGCGTACGCTGCAGGTCGAC -3'<br>5'- CTATTCATCAATAGGAATTTTAGGGACACTATGTGTACAAACTATC CGCTCAATCGATGAATTCGAGCTCG -3' | pFA6a–hphNT1 |

| | | |
|---|---|---|
| Control PCR *YOR019W* | 5'- CATTGATCTCGAGCACAGCTG -3'<br>5'- GTCGACCTGCAGCGTACG -3' | |
| *VCX1* deletion | 5'- ACGCATATCATTCATCGGCTGCTGATAGCAAATAAAACAACATAGA TACAATGCGTACGCTGCAGGTCGAC -3'<br>5'- GATAAAATATAAAAAAAAAGAGAATGGTGAATTTCTGCGCTACTGT TCTTACTCAATCGATGAATTCGAGCTCG -3' | pFA6a–hphNT1 |
| Control PCR *VCX1* | 5'- TAGATTGTTTTCCTTACGAC -3'<br>5'- GTCGACCTGCAGCGTACG -3' | |
| *RCN1* deletion | 5'- GGGCCAAAAAGATCAAGCAATAAACCAACCGATATATAAAACACAG AACTGCAGATGCGTACGCTGCAGGTCGAC -3'<br>5'- CCACCCGTAAGCATTTAAGTCTCTTAAGCCAACAAATCGCCTCGCC ATCTTAATCGATGAATTCGAGCTCG -3' | pFA6a–hphNT1 |
| Control PCR *RCN1* | 5'- GTAGCACATCTGCCATACTATC -3'<br>5'- GTCGACCTGCAGCGTACG -3' | |
| *RCN2* deletion | 5'- GCAATCAAAAACCAAAAAAAGATACATTAGCGTTAGAAAATCGGGA ATTATGCGTACGCTGCAGGTCGAC -3'<br>5'- CCCTTTTTCTGCTTTGTCTAATTTATAGTATTAGTTATGCTCTAGT GCTCTAATCGATGAATTCGAGCTCG -3' | pFA6a–hphNT1 |
| Control PCR *RCN2* | 5'- CTGTCGGTCTAATCTCGTTAATTG -3'<br>5'- GTCGACCTGCAGCGTACG -3' | |
| *ROD1* deletion | 5'- CGCGATTTCATCACACCATTCGCTTCTCTTCCTCCATAAGTAATAA TGCGTACGCTGCAGGTCGAC-3'<br>5'- GCTCATATCTTTTTGTCGAAATTTCGAAGACAAAAAATAAAGCAGT CTAATCGATGAATTCGAGCTCG -3' | pFA6a–hphNT1 |
| Control PCR *ROD1* | 5'- CAGTAGTCAGCGTTGGGCA -3'<br>5'- GTCGACCTGCAGCGTACG -3' | |
| *ROG3* deletion | 5'- CATAGAGGCAGCTCTCTTAGCAAAATAAAAATACAAAAAGTTCGAC ATGCGTACGCTGCAGGTCGAC -3'<br>5'- ATATGAAACTATACAAGCTTAATGCACGAGCCGAAACAATATCGGC GACTAATCGATGAATTCGAGCTCG -3' | pFA6a–hphNT1 |
| Control PCR *ROG3* | 5'- CTGCCTTCCGTACGTCACAA -3'<br>5'- GTCGACCTGCAGCGTACG -3' | |
| *SLM1* deletion | 5'- GAAAAAAAGTAGTTAACAATAAAAGAATACAACATTAAAAGAAAAA AAAATGCGTACGCTGCAGGTCGAC -3'<br>5'- ACATATGGATGGAAGGAAATAAAAACATTCTAGTTTACTATACTTG TATTTAATCGATGAATTCGAGCTCG -3' | pFA6a–hphNT1 |
| Control PCR *SLM1* | 5'- GTTCTCTTGGATGATCTGAC -3'<br>5'- GTCGACCTGCAGCGTACG -3' | |
| *SLM2* deletion | 5'- GGTTTAGGAGAGCGTTATACGCTTTACCCAGCCGTCCATTGCACCA TGCGTACGCTGCAGGTCGAC -3'<br>5'- CATAATATTACATACAAACTAGAGCATTACTATATAATTAACTTAC TTGTCAATCGATGAATTCGAGCTCG -3' | pFA6a–hphNT1 |
| Control PCR *SLM2* | 5'- CCGGCACTGCCTGCTTCTGT -3'<br>5'- GTCGACCTGCAGCGTACG -3' | |

| | | |
|---|---|---|
| *SPT8* deletion | 5’- GCAGTAACTGTAGTGTACTAAAGGCTCAGTTTTTTTTTTTTTTCTTC<br>TTTTACGTAATGCGTACGCTGCAGGTCGAC -3’<br>5’- GCGCGGAGTAATTATGATTATGATTATGGTTATGATTATTATTACA<br>ACTCACTAATCGATGAATTCGAGCTCG -3’ | pFA6a–hphNT1 |
| Control PCR *SPT8* | 5’- CGTATACACCCCGGTTGCCC -3’<br>5’- GTCGACCTGCAGCGTACG -3’ | |
| *UBX6* deletion | 5’- GACATTTTTGACCCTCAAAGGAAGTGAATTACAGGTATTGAATAAC<br>AGAAATGCGTACGCTGCAGGTCGAC -3’<br>5’- GAAAGAAAATATGTGTGAATAACCAAATAGGAAATAAACAAAAGCA<br>CATTTAATCGATGAATTCGAGCTCG -3’ | pFA6a–hphNT1 |
| Control PCR *UBX6* | 5’- CGAATCGTAACGGACCCAGC -3’<br>5’- GTCGACCTGCAGCGTACG -3’ | |
| *UBX7* deletion | 5’- GCACTTACAAAGAAGCAAGGATAGCTAAACCAAACAGCCGTAACCA<br>AATGCGTACGCTGCAGGTCGAC -3’<br>5’- CACGTCTAATACTAGTCATAAATGGAGTTGAAGCCATTAACTACTT<br>AATCGATGAATTCGAGCTCG -3’ | pFA6a–hphNT1 |
| Control PCR *UBX7* | 5’- CACCACCATCTGCCATCTCC -3’<br>5’- GTCGACCTGCAGCGTACG -3’ | |
| *YAP1* deletion | 5’- GTTTTTTGCCACCCAAAACGTTTAAAGAAGGAAAAGTTGTTTCTTA<br>AACCATGCGTACGCTGCAGGTCGAC -3’<br>5’- CATTATAGAAAAAGTTCTTTCGGTTACCCAGTTTTCCATAAAGTTC<br>CCGCTTTAATCGATGAATTCGAGCTCG -3’ | pFA6a–hphNT1 |
| Control PCR *YAP1* | 5’- CGGAAACGGCAGTAAACGAC -3’<br>5’- GTCGACCTGCAGCGTACG -3’ | |
| *CAD1* deletion | 5’- CTTTTAAGGACGACATATAAGCAATGGAACGACCAGTTAAGATAAA<br>TGCGTACGCTGCAGGTCGAC -3’<br>5’- CATGCTCTCATCCAACATTATGTATACTCAAGATATGTTTATATGC<br>TAATCGATGAATTCGAGCTCG -3’ | pFA6a–hphNT1 |
| Control PCR *CAD1* | 5’- GCAGCTATTGTTAAGGCGTC -3’<br>5’- GTCGACCTGCAGCGTACG -3’ | |
| *YPT6* deletion | 5’- GCTGTTGATTCTGAACAGTAAAAGATAAACAAAGAAGAGATTAACA<br>ATGCGTACGCTGCAGGTCGAC -3’<br>5’- CGCACACAAAGAGTTCTCCTTATGCCCTATAGAACTGAAATATTAG<br>GTGCTAATCGATGAATTCGAGCTCG -3’ | pFA6a–hphNT1 |
| Control PCR *YPT6* | 5’- CGTAATTACTATGTATAATGG -3’<br>5’- GTCGACCTGCAGCGTACG -3’ | |
| *PMR1* deletion | 5’- CAGCACAGACGTAAGCTTAAGTGTAAGTAAAAGATAAGATAA<br>TCAGCTGAAGCTTCGTACGC -3’<br>5’- TAACAGAGACAGTCCAACGGCGTAGTTGAACATTTTGTTG<br>CATAGGCCACTAGTGGATCTG -3’ | pUG6 |
| Control PCR *PMR1* | 5’- CTAGGCCATCGTACACTATAGC -3’<br>5’- GTCGACCTGCAGCGTACG -3’ | |
